# Human reference gut microbiome catalog including newly assembled genomes from under-represented Asian metagenomes

**DOI:** 10.1186/s13073-021-00950-7

**Published:** 2021-08-27

**Authors:** Chan Yeong Kim, Muyoung Lee, Sunmo Yang, Kyungnam Kim, Dongeun Yong, Hye Ryun Kim, Insuk Lee

**Affiliations:** 1 Department of Biotechnology, College of Life Science & Biotechnology, Yonsei University, Seoul, 03722 Korea; 2 Department of Laboratory Medicine, Research Institute of Bacterial Resistance, Yonsei University College of Medicine, Seoul, 03722 Korea; 3 Division of Medical Oncology, Department of Internal Medicine, Yonsei Cancer Center, Yonsei University College of Medicine, Seoul, 03722 Korea

**Keywords:** Metagenomic shotgun sequencing, Human gut microbiome, Metagenome-assembled genome, Cross-reactive antigen

## Abstract

**Background:**

Metagenome sampling bias for geographical location and lifestyle is partially responsible for the incomplete catalog of reference genomes of gut microbial species. Thus, genome assembly from currently under-represented populations may effectively expand the reference gut microbiome and improve taxonomic and functional profiling.

**Methods:**

We assembled genomes using public whole-metagenomic shotgun sequencing (WMS) data for 110 and 645 fecal samples from India and Japan, respectively. In addition, we assembled genomes from newly generated WMS data for 90 fecal samples collected from Korea. Expecting genome assembly for low-abundance species may require a much deeper sequencing than that usually employed, so we performed ultra-deep WMS (> 30 Gbp or > 100 million read pairs) for the fecal samples from Korea. We consequently assembled 29,082 prokaryotic genomes from 845 fecal metagenomes for the three under-represented Asian countries and combined them with the Unified Human Gastrointestinal Genome (UHGG) to generate an expanded catalog, the Human Reference Gut Microbiome (HRGM).

**Results:**

HRGM contains 232,098 non-redundant genomes for 5414 representative prokaryotic species including 780 that are novel, > 103 million unique proteins, and > 274 million single-nucleotide variants. This is an over 10% increase from the UHGG. The new 780 species were enriched for the *Bacteroidaceae* family, including species associated with high-fiber and seaweed-rich diets. Single-nucleotide variant density was positively associated with the speciation rate of gut commensals. We found that ultra-deep sequencing facilitated the assembly of genomes for low-abundance taxa, and deep sequencing (e.g., > 20 million read pairs) may be needed for the profiling of low-abundance taxa. Importantly, the HRGM significantly improved the taxonomic and functional classification of sequencing reads from fecal samples. Finally, analysis of human self-antigen homologs on the HRGM species genomes suggested that bacterial taxa with high cross-reactivity potential may contribute more to the pathogenesis of gut microbiome-associated diseases than those with low cross-reactivity potential by promoting inflammatory condition.

**Conclusions:**

By including gut metagenomes from previously under-represented Asian countries, Korea, India, and Japan, we developed a substantially expanded microbiome catalog, HRGM. Information of the microbial genomes and coding genes is publicly available (www.mbiomenet.org/HRGM/). HRGM will facilitate the identification and functional analysis of disease-associated gut microbiota.

**Supplementary Information:**

The online version contains supplementary material available at 10.1186/s13073-021-00950-7.

## Background

Human gut microbiome is considered the “second human genome” and plays a crucial role in various diseases [1, 2]. Therefore, targeting gut microbes and their functional elements may provide novel therapeutic opportunities. The assembly of human reference genome, together with a catalog of protein-coding genes and genomic variants, led us to the era of genomic medicine. Likewise, transformation of human medicine by harnessing the gut microbes requires the cataloging of reference microbial genomes and their encoded functional elements. Conventional approaches for microbial genome assembly require microbial isolation and culture. Indeed, with the development of culturomics technology, the number of culturable gut microbes has increased greatly [3–6]. However, the culturable taxa are biased towards specific clades, and a large portion of the human gut microbiome remains unculturable [7–9]. To address this, culture-independent methods of metagenome assembly from whole-metagenomic shotgun sequencing (WMS) data have been developed.

Recently, three independent studies have consecutively released large collections of prokaryotic genomes, including many based on metagenome assembly (Additional file 1: Table S1) [8–10]. The metagenome-assembled genomes (MAGs) from these studies were then combined with the genomic information deposited in public databases to generate integrated catalogs of prokaryotic genomes and proteins in the human gut [11], the Unified Human Gastrointestinal Genome (UHGG) and Unified Human Gastrointestinal Protein (UHGP) catalogs, respectively. The UHGG contains 204,938 non-redundant genomes that represent 4644 prokaryotic species and the UHGP catalogs approximately 95 million unique proteins.

Despite the latest advances, the current human gut microbiome catalog is incomplete, partially because the metagenome sampling is biased for geographical location and lifestyle. Specifically, the UHGG is strongly biased towards fecal samples collected in China, Denmark, Spain, and the USA. In the present study, to account for the under-sampling of certain metagenomes, we assembled genomes for fecal samples collected from Korea, India, and Japan. Since the genome assembly of low-abundance species in most human fecal samples may require a much deeper sequencing than usually employed, we performed ultra-deep WMS (> 30 Gbp or > 100 million read pairs) of 90 fecal samples collected from Korea. We also collected public WMS data for 110 and 645 fecal samples from India and Japan, respectively. We consequently assembled 29,082 prokaryotic genomes and combined them with the UHGG genomes to generate the Human Reference Gut Microbiome (HRGM), which substantially expands the list of representative species, genomes, proteins, and single-nucleotide variants (SNVs) in the human gut microbiome.

## Methods

### Whole-metagenome sequencing data for fecal samples from Korea, India, and Japan

We performed de novo genome assembly on WMS data for 90, 110, and 645 fecal samples from Korea, India, and Japan, respectively. WMS data for India and Japan populations were obtained from published but not included studies in the UHGG [12, 13]. Fecal WMS data for India were generated from 110 healthy donors in North-Central and Southern India [12]. Although the sequencing depth was relatively low (1.2 Gbp on average), it was expected that many novel genomes would be assembled because MAGs from India are not included in the existing catalogs. By contrast, 805 MAGs from Japan are included in the UHGG. However, it was expected that the inclusion of the recently published deep sequencing WMS data for 645 Japanese fecal samples (6.5 Gbp on average) [13] would greatly expand the number of MAGs for Japan. Ultra-deep WMS data (31 Gbp on average) were newly generated for fecal samples from 90 Koreans recruited at the Severance Hospital (Seoul, Korea). We collected fecal samples from donors using Norgen’s stool nucleic acid collection and preservation systems (Norgen Biotek Corp.; #63700). The UHGG does not contain any MAGs from Korea.

The libraries were prepared as described in the TruSeq Nano DNA Library Prep Reference Guide (Illumina #15041110). Briefly, 100 ng DNA was fragmented using LE220 Focused ultrasonicator (Covaris, Inc.). Fragmented DNA was end-repaired and approximately 350-bp fragments were obtained after size selection. After adapter ligation, eight PCR cycles were performed. Library quantification was performed as described in the Kapa Illumina Library Quantification Kit (Kapa Biosystems, #KK4854). Next, 150 bp × 2 paired-end sequencing was performed using Illumina HiSeq4000. In summary, new WMS data for 845 fecal samples collected from Korea, India, and Japan were obtained. The total read length was 7.2 Tbp. All samples used in the current study are described in Additional file 1: Table S2.

### Metagenome assembly and binning

The adapter sequences were trimmed, and low-quality bases and short reads were removed from WMS data using Trimmomatic v0.39 [14]. Next, the reads were aligned with the human genome GRCh38.p7 using Bowtie2 v2.3.5 [15], and the aligned reads were then removed. The majority of quality-controlled reads were assembled as contigs using metaSPAdes [16], which is a metagenome-specific pipeline of SPAdes v3.13.0. For unknown reasons, and regardless of sample size, metaSPAdes runtime was excessively long for 107 samples. In those cases, MEGAHIT v1.2.8 [17] was used (Additional file 1: Table S2).

Genome bins were generated using the ensemble approach and three binning tools: MetaBAT2 v2.13 [18], MaxBin2.0 v2.2.6 [19], and CONCOCT v1.1.0 [20]. First, the reads from each sample were first aligned with the assembled contigs from the previous step using Bowtie2, and the three binning programs were initiated. The minimum size of a contig for binning was set at 1000 bp, except for MetaBAT2, which requires at least 1500 bp. The three binning predictions were combined for improved binning results using the bin refinement module of MetaWRAP v1.2.2 [21], which uses CheckM v1.0.18 [22] to evaluate the quality of genome bins in terms of completeness and contamination rate. The minimum completeness was set at 50%, the maximum contamination at 5%, and the minimum quality score (*Completeness* − *5* × *Contamination*) at 50. The same threshold values for CheckM results were applied during the construction of the UHGG. This resulted in 7767 genomes from Korean samples, 563 genomes from Indian samples, and 20,752 genomes from Japanese samples (29,082 genomes in total).

### Generation of genomic species clusters

Groups of genomes that corresponded to species were generated using a two-step iterative procedure. Preliminary clustering was performed using Mash v2.2 [23] algorithm. Mash distances were calculated for all possible pairs of genomes using the “-s 10,000” parameter. Next, the average-linkage–based hierarchical clustering was performed, at a cutoff of 0.2. Mash algorithm is sufficiently fast to calculate all-by-all distances for hundreds of thousands of genomes in a timely manner. However, this compromises the accuracy, especially for low-coverage genome pairs [24], which are common in MAGs. Therefore, to improve cluster quality, average nucleotide identity (ANI) was calculated for every pair of genomes within each initial cluster by ANImf [24]. To avoid the overestimation of ANI by local alignment, a minimum coverage threshold was applied for each pair. The coverage cutoff of genome A and genome B was determined at *min(0.8, Completeness of genome A* × *Completeness of genome B)*. If the alignment coverage between two genomes was lower than the cutoff, they were regarded as different genomes. The genomes were then clustered using the average-linkage–based hierarchical clustering at a cutoff of 0.05 (or 95% identity), which is a widely accepted ANI threshold for species-level boundary [4, 9–11, 25]. The genome intactness score (*S*) [9, 11], *S = Completeness* − *5* × *Contamination* + *0.5* × *log*_*10*_*(N50)*, was then calculated. For clusters containing more than two genomes, a genome with the highest *S* was selected as the representative genome for the cluster. The above two-step procedure was iterated until the clusters ceased to change. Hence, 2199 species clusters were generated for 29,082 genomes from KIJ samples, with eight iterations of the aforementioned procedure. Finally, the 2199 genomes were combined with 4644 genomes from the UHGG, generating 5414 species clusters for the HRGM at the fourth iteration.

### Assessment of genome quality

The assembled microbial genomes were classified into three categories: high-quality (HQ), genomes with 5S, 16S, 23S rRNA, ≥ 18 tRNAs, ≥ 90% completeness, and < 5% contamination; near complete (NC), genomes with ≥ 90% completeness and < 5% contamination; and medium quality (MQ), genomes with 50 ~ 90% completeness and < 5% contamination. We used barrnap v0.9 [26] software for annotating the 5S, 16S, and 23S rRNA. We applied the “--evalue 1e-05” parameter, and “--kingdom bac” and “--kingdom arc” parameters for bacterial and archaeal genomes, respectively. We searched for tRNA with tRNAscan-SE v2.0.7 [27] with “-B” option for bacterial genomes and “-A” options for archaeal genomes. Genome quality of the 5414 representative species are summarized in Additional file 1: Table S4.

To evaluate the novelty of the new species generated from KIJ samples, we estimated ANI of their representative genomes to the UHGG genomes. Because calculating all pairwise ANIs takes a huge computational cost, we calculated ANImf for the 10 closest UHGG species representative genomes from each of the novel species representative genomes based on the IQ-tree distance, which is based on the concatenated sequence of 120 marker genes.

We calculated strain heterogeneity as described in previous studies [10, 11]. Briefly, we aligned sequencing reads of the originated sample against MAGs using Bowtie2, and the alignment results were sorted and indexed with Samtools [28]. Next, the number of non-synonymous and synonymous mutations was calculated with the polymut.py script of CMSeq package [10]. We used “--mincov 10” and “--minqual 30” parameters for concordance with previous analyses [10, 11]. The number of non-synonymous mutations divided by the total number of considered positions was used as the strain heterogeneity of a genome.

### Non-redundant genome counting

To count the number of non-redundant genomes, the redundant genomes were removed, similar to what was done for the UHGG pipeline [11]. First, the pairwise genome distance was calculated using Mash [23] and the entire genomes were clustered using average-linkage–based hierarchical clustering, with a 0.001 cutoff (Mash ANI 99.9%). To reduce the computation time, the hierarchical clustering was performed only for the connected components with the distance of 0.1, because it is highly unlikely that genomes that are not within the distance of 0.1 are clustered together by a distance of 0.001. In the process, 22,761 genomes were clustered into 8508 conspecific clusters. Multiple genomes from the same sample for the same species cluster were counted only once.

### Taxonomic and functional annotation of representative species genomes

The taxonomic annotation of 5414 representative species genomes was performed using the “classify_wf” function of GTDB-Tk v1.0.2 [29]. The reference version was GTDB R04-RS89, released in June 2019. Genomic features, such as CDS, rRNA, and tRNA, were identified and annotated in each genome using Prokka v1.14.5 [30] with “--kingdom Bacteria” and “--kingdom Archaea” parameters for the bacterial and archaeal genomes, respectively. With the protein sequences predicted by Prokka, the antibiotic resistance genes were annotated using RGI v5.1.0 [31] with default parameters. Finally, the secondary metabolite gene cluster was annotated using antiSMASH v5.1.2 [32]. For the full-featured annotation, the “--cb-general, --cb-knownclusters, --cb-subclusters, --asf, --pfam2go, --smcog-trees, --cf-create-clusters” parameters were set.

To render the HRGM useful for the 16S rRNA sequencing–based metagenomic analysis, the 16S rRNA regions for 5414 representative species genomes were predicted using barrnap v0.9 [26] tool and the “--evalue 1e-05” parameter, and “--kingdom bac” and “--kingdom arc” parameters for bacterial and archaeal genomes, respectively. The 16S rRNA sequences were thus directly predicted from 1364 representative species genomes. For the remaining 4050 representative species, the search for 16S rRNA sequences was expanded to their conspecific genomes. The barrnap analysis was used for the genomes from KIJ samples and pre-established 16S rRNA region annotations were used for the genomes from the UHGG. Within the expanded search space, 16S rRNA sequences were identified for 1178 additional genomes. Consequently, 16S rRNA sequences were generated for 2542 species in the HRGM.

### SNV analysis

For the species clusters with more than three genomes, SNVs were identified using the codes provided by the UHGG [11]. Briefly, every non-representative genome, *g*, was aligned with the representative genome, *r*, in the species cluster using nucmer 4.0.0beta2 [33]. Best bi-directional alignments were identified using the delta-filter program and “-q –r” options, and SNVs were annotated using the show-snp program; nucmer, delta-filter, and show-snp are software packages of MUMmer v3 [34]. For each species cluster (*G*) whose representative genome is *r*, we calculated the SNV density with normalization of the number of SNV by the aligned genome length and the number of genomes in the *G*.

$$ SNV\  per\  kb=\frac{\sum \limits_{g\in \left(G-\left\{r\right\}\right)}\frac{\#{SNV}_{r,g}\ }{{Aligned\ length}_{r,g}/1000}}{n(G)-1} $$

*SNV per kb* was only calculated for 1521 species clusters with ≥ 10 genomes to reduce sampling bias. For the 1521 genomes, the average phylogenetic distance to the five nearest species was calculated using the IQ-Tree [35].

To evaluate the normality of SNV frequency across genomic regions for the 1521 *G*s, we counted the number of SNV in the chunk (fragment) of the aligned region between *r* and *g*. We used 50-kb and 100-kb chunks for the *r* with *SNV per kb* > 5 and *SNV per kb* ≤ 5, respectively. The normality of the number of SNVs per chunk was tested for each *r–g* pair using Kolmogorov*–*Smirnov test.

### Cataloging gut prokaryotic proteins and their functional annotation

Overall, 64,661,728 CDS were identified in 29,082 genomes from the KIJ samples using Prodigal v2.6.3 [36] and “-c -m -p single” parameters. Since many proteins were derived from conspecific genomes, the catalog may have included many homologous proteins. To reduce the redundancy in the protein catalog, CD-HIT v4.8.1 [37] was adopted. To reduce CD-HIT running time, identical proteins were first clustered and then CD-HIT was executed at 100% similarity level. The cataloged proteins were then combined with those in UHGP-100 [11]. The consolidated protein catalog was subsequently submitted to CD-HIT clustering analysis at five different sequence similarity levels, 100%, 95%, 90%, 70%, and 50%. For accurate and efficient clustering, a multi-step iterative clustering method recommended by the CD-HIT tutorial was adopted. For instance, the CD-HIT-95 protein catalog (a 95% similarity level protein catalog) was constructed based on CD-HIT-100 proteins, and the CD-HIT-90 protein catalog was constructed based on CD-HIT-95 proteins. This resulted in approximately 103.7 million, 20.0 million, 14.8 million, 8.5 million, and 4.7 million proteins at the sequence similarity levels of 100%, 95%, 90%, 70%, and 50%, respectively.

Representative protein sequences in the five protein catalogs were functionally annotated using eggNOG-mapper v2.0.1 [38], which is based on the eggNOG protein database v5.0 [39]. The resultant annotations include eggNOG orthologs and functional terms from several databases, including Gene Ontology (GO) [40] and Kyoto Encyclopedia of Genes and Genomes (KEGG) [41]. Further, for each protein cluster, taxonomic origins of all member proteins and the lowest common ancestor of the cluster were tracked and annotated.

The numbers of shared species and shared phyla of proteins in the HRGM-50 protein catalogs were annotated based on the taxonomic annotation of member proteins. The number of shared species was binned at the bin size of 10, then the annotation rate for each protein bin was calculated as the number of annotated proteins divided by the number of proteins in the bin.

### Reconstruction of the phylogenetic tree

For the bacterial and archaeal genomes, 120 and 122 universal marker genes, respectively, were predicted by the GTDB-Tk [29]. Using the concatenated sequences of marker genes, the maximum-likelihood tree was generated using IQ-TREE [35]. The phylogenetic tree of bacterial genomes was visualized using iTOL [42].

### Kraken2 databases

The Kraken2 v2.0.8-beta [43] custom database for the HRGM representative genomes was prepared based on the taxonomic annotations in GTDB-TK [29]. When two or more genomes were annotated to the same taxon, they were discriminated at the succeeding lower rank. For example, if *genome a* and *genome b* were both annotated to *species_A*, *genome a* and *genome b* were annotated as *Species_A;strain_1* and *Species_A;strain_2*, respectively. By doing so, the user can select a taxonomic rank, thereby measuring species abundances together or individually. The Kraken2 database for the UHGG [11] was downloaded from UHGG FTP on March 6, 2020. The Kraken2 standard database was downloaded and constructed using “kraken2-build --standard” command on July 14, 2020.

### Measuring taxonomic classification rate of sequencing reads

WMS data were compiled for publicly available data for 926, 54, and 26 fecal samples from the USA [44], Cameroon [45], and Luxembourg [46, 47], respectively. WMS data for 16 fecal samples collected from Korea, which were not included in the HRGM, were also used. These 1022 fecal samples were neither used for the UHGG nor for the HRGM. The data were pre-processed and taxonomically classified using Kraken2 with standard database, UHGG-based database containing 4644 representative genomes, and HRGM-based database containing 5414 representative genomes. The taxonomic classification rate was then calculated based on the proportion of aligned sequence reads in a sample with respect to the database.

### Measuring functional classification rate of sequencing reads

The functional classification rate of sequencing reads was determined based on the number of aligned reads against the protein catalog. For the analysis, WMS data were randomly selected for ten fecal samples from each of the Cameroon, Korea, USA, and Luxembourg cohorts (the same cohorts were used for the measuring taxonomic classification rate). After pre-processing, 40 samples were aligned with the UHGP-95 and HRGM-95 protein databases using blastx of DIAMOND v0.9.35.136 [48]. The results were filtered at > 80% query coverage (read coverage) and > 95% alignment identity thresholds. A pair of reads was treated as two independent reads. For multiple alignments of a read, only the best alignments by bit score and *E*-value were considered.

### Evaluation of the effect of sequencing depth on de novo genome assembly

Nine Korean samples with sequencing depth of > 52.5 Gbp (Additional file 1: Table S2) were selected for analysis. Then, 0.5, 2.5, 5, 10, 20, 40, 80, 125, and 175 million read pairs were randomly sampled from each of these samples. As the average read-pair length was 300 bp, the sequencing depths of these random samples corresponded to 150 Mbp, 750 Mbp, 1.5 Gbp, 3 Gbp, 6 Gbp, 12 Gbp, 24 Gbp, 37.5 Gbp, and 52.5 Gbp, respectively (Additional file 2: Fig. S2). For the 81 simulated samples (9 samples × 9 depths), de novo genome assembly was performed using the same pipeline as that used for the database construction.

Two adjacent sequencing depths (e.g., 125 *vs.* 175 million read pairs) were compared to evaluate the effect of sequencing depth on the de novo genome assembly. Samples with a greater sequencing depth may yield more MAGs with over 50% completeness, yet with a lower average quality than those with a lower sequencing depth because of MAGs that barely pass the completeness threshold. Therefore, instead of the average quality scores of all assembled genomes, two genomes assembled at different sequencing depths for the same species clusters were compared. Mash [23] clustering of genomes from two random samples was performed for a comparison based on the average-linkage–based hierarchical clustering, at a threshold of 0.1 (90% identity). Mash clustering was sufficient for clustering conspecific genomes in the simulated samples. Indeed, no cluster had more than two genomes from the same sequencing depth. The assembly quality (completeness, contamination, N50, and genome size) of conspecific genomes at adjacent sequencing depths was then compared.

### Evaluation of the effect of sequencing depth on taxonomic profiling

To avoid overestimation of performance, WMS data for 16 Korean fecal samples that have not been used for the HRGM construction and generated at a sequencing depth of > 24.5 Gbp were used. From each of the 16 samples, 1, 5, 10, 20, 40, 60, and 80 million read pairs that corresponded to 300 Mbp, 1.5 Gbp, 3 Gbp, 6 Gbp, 12 Gbp, 18 Gbp, and 24 Gbp, respectively, were randomly sampled. Taxonomic profiling was then conducted using Kraken2 and the HRGM-based database. Based on the hypothesis that profiling of low-abundance taxa is more affected by sequencing depth than abundant ones, the taxonomic features were stratified at eight different levels of relative abundance, ranging from 1e−07 to 1 with every ten-fold increase. Pearson correlation coefficient (*PCC*) and Spearman correlation coefficient (*SCC*) between the taxonomic profiles at different sequencing depths were then calculated for each group of features for different levels of relative abundance.

### Analysis of cross-reactivity potential of microbes and their association with diseases

Epitope sequences from autoimmune disease-related self-antigen were compiled from the Immune Epitope Database (IEDB) [49]. “Epitope: Linear epitope”, “Antigen: Organism: Homo sapiens”, “Host: Homo sapiens”, and “Disease: Autoimmune Disease” filters of the IEDB web portal were applied. Epitope sequences that required post-translational modification (e.g., citrullination and deamination) and epitopes shorter than five amino acids were ignored. Next, 24,461 unique epitope sequences were aligned with the protein sequences encoded by 5414 species representative genomes using BLASTP [50]. For meticulous alignment of short peptide sequences, “-word_size 4”, “-evalue 10000”, and “-max_target_seqs 100000” options were applied. For every epitope-to-gene pairwise alignment, the Alignment Score (*AS*) was calculated, as follows:

*AS* = (match length - gap length) / epitope length

Alignments with *AS* = 1 were used to count the number of epitope-containing genes (ECGs) for every representative species. The number of ECGs was positively correlated with the number of genes of the species genomes. Therefore, the number of ECGs was normalized by the total number of genes for each species genome. To identify epitope-enriched taxonomic clades, ECGs per gene of each taxonomic group were compared with the entire 5414 genomes, and Mann–Whitney *P* values and fold-change were calculated. We identified trans-membrane helices (TMHs) and signal peptides of the ECGs using the TMHMM v2.0c and SignalP v5.0b, respectively. We ran TMHMM with default parameters and SignalP with -org arch for archaeal proteins and applied both “-org gram+” and “-org gram-” parameters for bacterial proteins.

To systematically evaluate associations of ECG-enriched taxa with human gut microbiome-associated diseases, we compiled taxon-disease pairs annotated by gutMDisorder database [51] as of May 2021. We defined high cross-reactivity taxa by ECG count enrichment (*P* < 1e−05) and then included the child taxa for the following ECG-enriched taxa: *Atopobiaceae* (level: family, NCBI txid: 1643824), Bacteroidia (class, 200643), *Bifidobacteriaceae* (family, 31953), *Oscillospiraceae* (family, 216572), *Sutterella* (genus, 40544), and Verrucomicrobiota (phylum, 74201). The taxa without NCBI txid were manually classified. All other taxa were defined as low cross-reactivity taxa. We tested associations of the high cross-reactivity taxa for the diseases with more than 40 annotated taxa using gutMDisorder. The associations were assessed by odds ratio (OR) (odds that high cross-reactivity taxa are annotated for the disease / odds that low cross-reactivity taxa are annotated for the disease) and their significance by Fisher’s exact test. Here, OR > 1 indicates that high cross-reactivity taxa are more associated with the disease than low cross-reactivity taxa are. The direction of association was also assessed by OR (odds that high cross-reactivity taxa are annotated to increase in the disease / odds that low cross-reactivity taxa are annotated to increase in the disease) and their significance by Fisher’s exact test. Here, OR > 1 indicates that high cross-reactivity taxa tend to increase in the disease.

## Results

### Assembly of gut microbial genomes from Korea, India, and Japan

We assembled prokaryotic genomes using an in-house bioinformatics pipeline (Additional file 2: Fig. S1a, Methods), which is more exhaustive than similar approaches [8–11] (Additional file 1: Table S1). For instance, we adopted an ensemble method for binning assembled contigs, as it showed better performance than individual binning tools [21, 52]. We hypothesized that metagenomes harbored by individuals from under-represented geographical locations and lifestyles would expand the current catalog of human gut microbiome. Therefore, we performed de novo genome assembly of fecal samples from three Asian countries: Korea, India, and Japan (referred to here as KIJ samples, Additional file 1: Table S2). At the start of the current study, WMS data for 645 and 110 fecal samples from Japan and India, respectively, were publicly available but not included in the UHGG [12, 13]. To complement these public data, we generated additional WMS data for fecal samples collected from 90 donors recruited in Korea. We set the minimum completeness at 50%, the maximum contamination at 5%, and the minimum quality score (*Completeness* − *5* × *Contamination*) at 50 for genomes of minimum quality. This yielded 29,082 MAGs: 7767 from Korea, 563 from India, and 20,752 from Japan.

### Ultra-deep sequencing facilitates the genomic assembly of low-abundance taxa

**Fig. 1:**
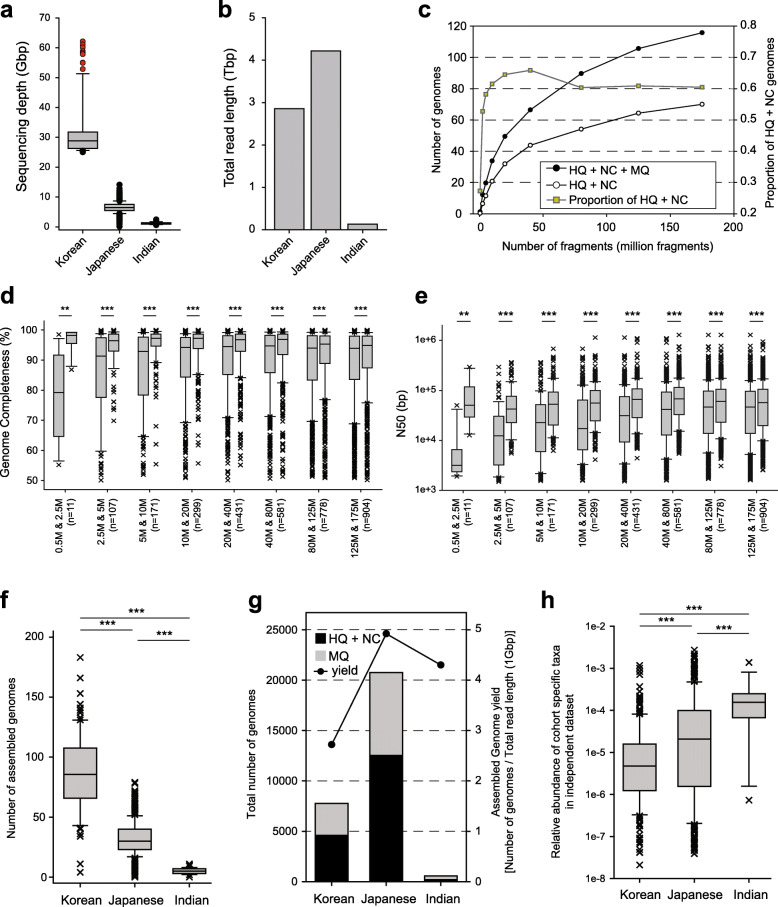
Effect of sequencing depth on de novo genome assembly. **a** Sequencing depth of samples from Korea, Japan, and India. Red data points, nine samples used for the generation of simulated samples for different sequencing depths. **b** Total read length of samples from Korea, Japan, and India. **c** The average number of genomes (left axis) and the proportion of (HQ + NC) genomes (right axis) from nine samples. **d, e** Completeness (**d**) and N50 (**e**) of assembled genomes from lower sequencing depth (left box of each column) and greater sequencing depth (right box of each column). **f** The number of the assembled genomes from Korea, Japan, and India. **g** Total number of the assembled genomes from Korea, Japan, and India, and genome assembly yields. **h** The relative abundance of 224 Korea-specific, 338 Japan-specific, and 18 India-specific assembled genomes in independent fecal samples from the USA (*n* = 926). *P* values were calculated by two-sided Mann–Whitney *U* test (**: *P* < 0.01; ***: *P* < 0.001).

To investigate the impact of metagenome sequencing depth on de novo genome assembly, we performed ultra-deep sequencing of the 90 Korean fecal samples (> 30 Gbp or > 100 million read pairs); the depth was approximately 5-fold deeper than the normal sequencing depth (Fig. 1a). Despite sequencing at the normal depth, fecal samples from Japan had a larger total read length than Korean samples because of a much larger sample size (Fig. 1b). For nine of the 90 Korean samples, approximately 60 Gbp was sequenced for the study of sequencing depth effect on genome assembly. We then generated 81 simulated WMS datasets (9 different depths for each of the 9 original samples with ~ 60 Gbp depth) and used the same pipeline of de novo genome assembly for all samples. As expected, the number of all MAGs with minimum quality (HQ + NC + MQ) increased with the increasing sequencing depth. However, the growth rate simultaneously decreased and the proportion of (HQ + NC) MAGs became stable after the initial phase of rapid growth (Fig. 1c). Next, we investigated whether the increased sequencing depth improved the quality of MAGs. We compared the assembly quality of MAGs for the same species in two different simulated samples at adjacent sequencing depths (Additional file 2: Fig. S2, Methods). The quality of MAGs from the greater sequencing depth was significantly higher than that of genomes from the lower sequencing depth in terms of completeness, contamination, N50, and genome size (Fig. 1d, e, Additional file 2: Fig. S3a-b). However, the degree of improvement of the assembly quality diminished as the sequencing depth increased.

We then examined the effect of sequencing depth using the actual WMS data for KIJ samples. The number of all MAGs with minimum quality from each cohort was the highest in the ultra-deep sequenced samples from Korea (Fig. 1f). However, the proportion of (HQ + NC) MAGs in samples from Korea and Japan was not significantly different (Fig. 1g; Additional file 2: Fig. S3c). Notably, the genome assembly yield, i.e., the number of assembled genomes divided by the total sequencing length, was highest for samples from Japan (Fig. 1 g). This suggests that sequencing hundreds of samples at a depth of 5–10 Gbp may constitute the most effective strategy for cataloging MAGs for a given population.

The ultra-deep sequencing may be advantageous for the genome assembly for low-abundance taxa. To test this, we used representative MAGs for the species clusters composed of MAGs from exclusively one of the three Asian countries and not included in the UHGG, i.e., representative MAGs for 224, 388, and 18 species clusters from Korea, Japan, and India, respectively. Therefore, these representative species MAGs were specific to each country. The average completeness and contamination of these country-specific representative species MAGs were 86.06% and 1.4%. We then estimated their relative abundance in fecal samples in an independent population of 926 fecal samples from the USA [44] using Kraken2 [43]. The Korea-specific representative species MAGs shifted towards low-abundance taxa compared with representative species MAGs specific to other countries (Fig. 1h), which supports our hypothesis. We also found that some family taxa were enriched or depleted among the MAGs specific to each country (Additional file 1: Table S3, *P* < 0.05 by Fisher’s exact test).

### Cataloging reference genomes of 5414 prokaryotic species from the human gut

**Fig. 2:**
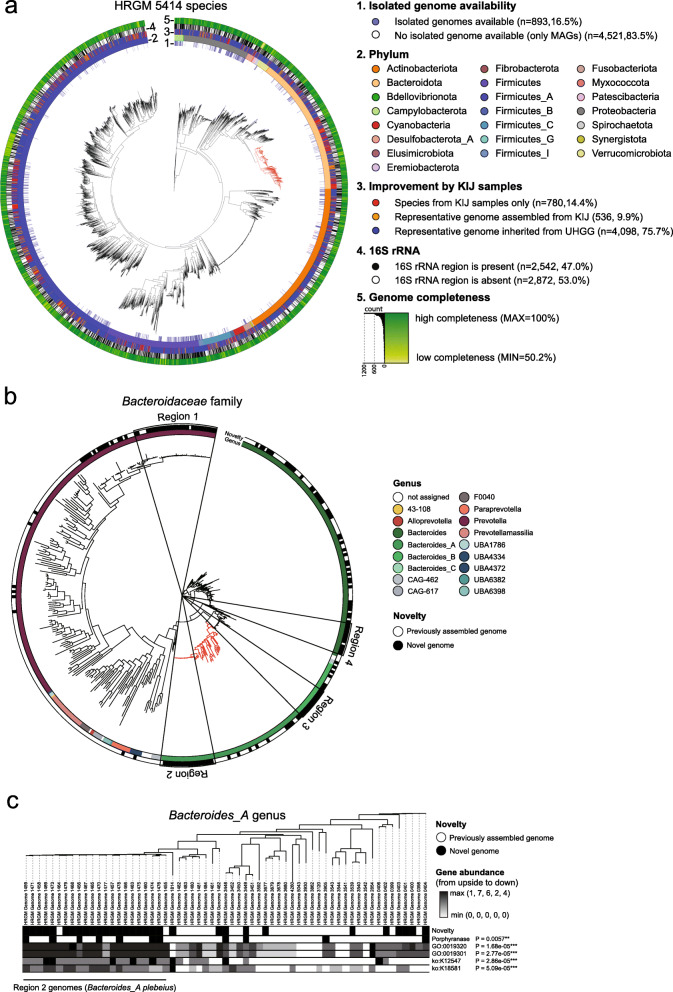
Phylogenetic trees of 5386 representative species of HRGM and species from KIJ samples. **a** Maximum-likelihood phylogenetic tree reconstructed from 120 bacterial marker genes (“Methods”). Representative genomes were annotated by their isolated genome availability (1st layer from the inside), phylum classification (2nd layer), whether they were from UHGG or assembled from KIJ samples (3rd layer), 16S rRNA sequence availability (4th layer), and genome completeness (the outermost layer). Red branches represent 410 species belonging to *Bacteroidaceae* family that are enriched in the representative species updated by including KIJ samples. **b** *Bacteroidaceae* family includes many new species from KIJ samples. The inner color strip indicates genus classification by GTDB-TK, and the outer color strip marks novel genomes from KIJ samples. Region 1 belongs to *Prevotella* genus and includes 30 species of *Prevotella copri*. Region 2 belongs to *Bacteroides_A* genus and includes 22 species of *Bacteroides_A plebeius*. Red branches indicate *Bacteroides_A* genus. Region 3 belongs to *Bacteroides_B* genus and 12 species of the region are annotated as *Bacteroides_B vulgatus*. Region 4 includes *Bacteroides* genus and all 13 species are classified as *Bacteroides xylanisolvens* by GTDB-TK. **c** Phylogenetic tree of 61 species of *Bacteroides_A* genus, the red branches in **b**. The uppermost color strip represents the novel species from KIJ samples. The second strip indicates the species harboring Porphyranase gene. Other gradient bars indicate the abundance of the gene with corresponding functional categories. *P* values indicate the statistical significance of enrichment of the function for the novel genomes compared with others in the *Bacteroides_A* genus. *P* values were calculated by the two-sided Fisher’s exact test (for the presence of Porphyranase) or two-sided Mann–Whitney *U* test (for abundance functional categories).

To construct the most comprehensive reference database for the human gut microbiome, we integrated the newly generated 29,082 MAGs from KIJ samples with the UHGG genomes using dereplication approach (Additional file 2: Fig. S1b, Methods). Dereplication of the 29,082 MAGs resulted in 2199 clusters of genomes. We selected a representative genome from each cluster to catalog the genomes for 2199 representative species, which we then integrated with 4644 representative genomes from the UHGG, via dereplication, resulting in 5414 clusters of genomes. Finally, we selected 5414 representative genomes and assigned their phylogenetic classifications using GTDB-Tk [29] (Fig. 2a, Additional file 1: Table S4). Based on the three categories of assembled genome quality (“Methods”), we found 763 (14.1%), 2933 (54.2%), and 1718 (31.7%) representative genomes belonging to HQ, NC, and MQ categories, respectively. Among these representative genomes, 4531 (83.7%) genomes were exclusively assembled from metagenomic data, which confirmed the notion that the major portion of the human gut microbiome has not yet been isolated. We identified 16S rRNA sequences in 2542 representative genomes (47%) (Additional file 2: Fig. S4), covering the majority of phylogenetic clades.

The inclusion of MAGs from KIJ samples in the new database allowed several improvements from the UHGG. First, we reduced the data bias towards China among Asian countries (Additional file 2: Fig. S5a). Second, we expanded the total number of non-redundant reference genomes by 13.25% and the number of representative species by 16.6% (Additional file 1: Table S5). Among the 5414 species clusters, 780 (14.4%) were generated by KIJ samples only, and representative genomes for 536 species clusters of the UHGG (9.9%) were replaced with the new MAGs from KIJ samples. Hence, in total, 1316 representative genomes (24.3%) were updated in the HRGM. The remaining 4098 species clusters (75.7%) inherited their representative genomes from the UHGG (Fig. 2a, Additional file 2: Fig. S5b). To evaluate whether the 780 novel species were based on spurious clusters or not, we examined the ANI of their representative genomes to the UHGG representative species genomes. We found that most of the 780 novel species by HRGM showed ≤ 0.1, and none of them showed ≥ 0.95 for mean ANI to the 10 closest UHGG species representative genomes (Additional file 2: Fig. S5c). We also found that only 15 out of the 780 species (1.92%) had maximum ANI > 0.95, indicating that most of the species fulfill the criterion of species discrimination. This small fraction of spurious novel species might be due to the widely used two-step clustering procedure for genome dereplication that includes preliminary clustering using Mash algorithm [23]. Therefore, we conclude that most of the 780 species clusters generated from KIJ samples represent genuinely novel species.

### New species from Korea, India, and Japan are associated with diet-related lifestyles

Notably, *Bacteroidaceae* family (red tree branches in Fig. 2a) was enriched in the new species (*P* < 0.001, Fisher’s exact test). Almost half the species from this family are from the *Bacteroides* genus and approximately two-thirds of the other half are from the *Prevotella* genus (Fig. 2b). Functional enrichment analysis for the new species belonging to these genera (Additional file 1: Table S6-7) revealed that new species belonging to the *Bacteroides* genus are enriched for enzymes involved in carbohydrate metabolism. Interestingly, four regions in the phylogenetic tree were highly enriched in the new species. The “region 1” encompasses a portion of the *Prevotella* genus and includes 30 species annotated as *Prevotella copri*. Previously, westernized populations with a typically high-fat and low-complex carbohydrate diet were reported to exhibit low prevalence and diversity of *P. copri* compared with non-westernized populations [53]. The “region 2” encompasses a portion of the *Bacteroides_A* genus and includes 22 species annotated as *Bacteroides_A plebeius*. It has been suggested that *Bacteroides plebeius* harbors genes encoding an enzyme specific for algal carbohydrates, acquired from marine microbes [54]. This species is typically found in Japanese subjects whose diet includes seaweed-rich food, such as sushi. We found that most of the 22 species belonging to *Bacteroides_A plebeius* contain homologous genes for porphyranase, an enzyme responsible for the degradation of porphyrin, which composes the cell wall of red algae (Fig. 2c, Additional file 1: Table S8). We also found that 21 out of 22 species belonging to *Bacteroides_A plebeius* were assembled from samples from Japan. These results validate the previous report of the association between gut bacterium *Bacteroides plebeius* and Japanese lifestyle. Not only the enzyme porphyranase but also many functions involved in carbohydrate metabolism were found to be enriched among 61 species belonging to *Bacteroides_A* genus (Additional file 1: Table S9). The “region 3” encompasses a portion of the *Bacteroides_B* genus and includes 12 species annotated as *Bacteroides vulgatus*, which is typically present in the human distal gut, where undigested plant polysaccharides and proteins exist in large quantities [55]. The “region 4” encompasses a portion of the *Bacteroides* genus and includes 13 species annotated as *Bacteroides xylanisolvens*, a xylan-degrading bacterium isolated from human faces [56]. Together, these observations suggest that the new species from KIJ samples are associated with the diet-related lifestyles.

### Identification of subspecies clades that are endemic to specific East Asian countries

Owing to the substantially increased coverage of gut microbial genomes for two East Asian countries, Korea and Japan, we had the opportunity to investigate whether there are gut bacterial subspecies genomes that are endemic to specific East Asian countries. We first selected HQ and NC genomes that have been assembled from four East Asian countries (Korea, Japan, China, and Mongolia) for the 74 species clusters with more than 1000 member genomes. For the selected genomes, we extracted 120 bacterial marker genes and performed multiple sequence alignment with the align module of GTDB-TK. Next, we generated the maximum-likelihood phylogenetic tree using IQ-Tree with default parameters and visualized tree with country annotation with iTOL. Through visual inspection of the subspecies trees for the 74 species, we identified two species with distinct subspecies clades that are endemic to specific countries. We observed a subspecies clade of *Escherichia coli D* that is endemic to Japan (Additional file 2: Fig. S6a). We verified that the most enriched KEGG ortholog for this subspecies clade of the species was mhqR, a multiple antibiotics resistance regulator (MarR) (Additional file 1: Table S10) [57]. We also observed a subspecies clade of *Bifidobacterium adolescentis* that is endemic to Mongolia (Additional file 2: Fig. S6b). These subspecies were found to be depleted in KEGG orthologs for type IV pilus assembly proteins and various transporters, both of which are potentially associated with antibiotic resistance (Additional file 1: Table S11). Notably, the endemic subspecies clade to Mongolia has higher abundance of genes for β-galactosidase, also called lactase, compared with other subspecies. These subspecies of *Bifidobacterium adolescentis* with high potential of lactase production can be attributable to the Mongolian cuisine, which predominantly consists of dairy products. Taken these results together, utilizing expanded genome catalogs by new MAGs, we could demonstrate that there are gut bacterial subspecies endemic to different countries within geographic regions in Asia.

### SNV density is positively associated with the speciation rate of gut commensals

**Fig. 3:**
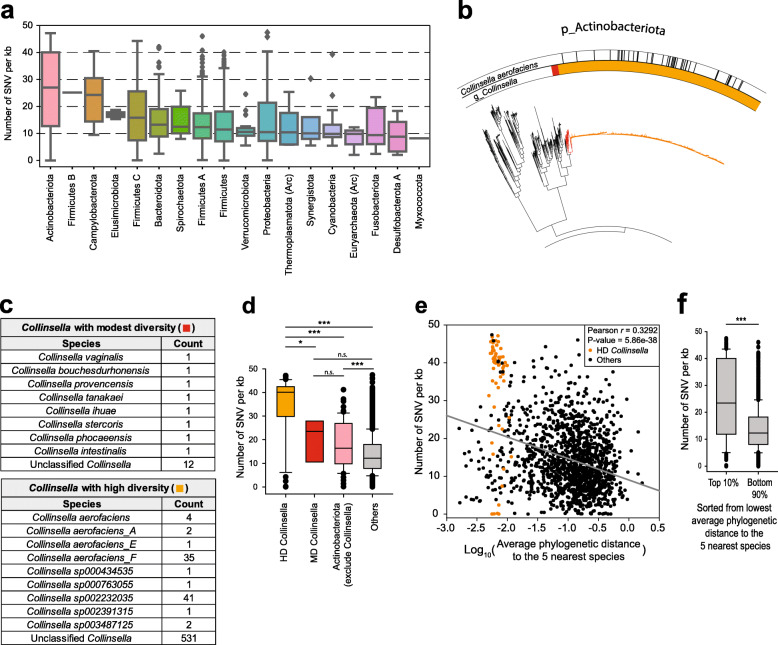
SNV density analysis of the relationship between within-species variation and speciation of gut microbes. **a** The number of SNVs per kb pair of the aligned region. SNV density is summarized for each phylum. Boxes are sorted by the median. Arc, archaeal phylum. **b** The phylogenetic tree for Actimobacteriota phylum. Inside annotation indicates the *Collinsella* genus, divided into *Collinsella* with modest phylogenetic dispersion (MD *Collinsella*, Red) and *Collinsella* with high phylogenetic dispersion (HD *Collinsella*, Orange). Black annotations in the outer circle represent *Collinsella aerofaciens*, *Collinsella aerofaciens_A*, *Collinsella aerofaciens_E*, and *Collinsella aerofaciens_F*, according to the GTDB-TK annotation. **c** GTDB-TK based taxonomic annotation of MD *Collinsella* and HD *Collinsella*. **d** SNV density of HD *Collinsella*, MD *Collinsella*, Non-*collinsella* actinobacteriota, and other species. **e** Scatter plot analysis of SNV density of 1521 representative species with ≥ 10 subspecies genomes in the cluster and their average phylogenetic distance to the five nearest species of each representative species. Orange points denote species of HD *Collinsella* and black points represent other species. **f** Comparison of SNV density between the top 10% and bottom 90% of the 1521 species sorted from the lowest average phylogenetic distance to the five nearest species. Statistical significance was calculated by two-sided Mann–Whitney *U* test (n.s.: not significant; *: *P* < 0.05; ***: *P* < 0.001)

We then aligned genomes of species clusters containing ≥ 3 genomes with the representative genome and mapped SNVs (“Methods”). This yielded 274,543,071 SNVs from 2821 species clusters, representing 10.07% and 13.34% increases, respectively, from the UHGG. We calculated SNV density across genomes based on *SNV per kb* for only 1521 species clusters with ≥ 10 genomes to reduce sampling bias. The Actinobacteriota phylum had the highest SNV density (Fig. 3a). Parts of a genome with a relatively higher level of SNV density would suggest possible chimerism. To test whether the SNVs were evenly distributed across the genome, we investigated the distribution of the number of SNVs per chunk (fragments of 50 kb or 100 kb) for the 1521 species clusters (“Methods”). There were 304,769 representative genome and non-representative genome (*r–g*) pairs with ≥ 5 chunks. If SNV density is significantly affected by the chimeric region, the distribution of the number of SNV per chunk may not follow a normal distribution. We found that most of the *r–g* pairs (300,545/304,769, 98.61%) followed a normal distribution (Kolmogorov*–*Smirnov test, *q*-value > 0.05) (Additional file 1: Table S12 and Additional file 2: Fig. S7), which suggests that the level of chimerism is low for most of the assembled genomes.

Phylogenetically overdispersed branches of Actinobacteriota phylum were apparent in both, the HRGM and UHGG. The majority of genomes from the overdispersed tree region belonged to the *Collinsella* genus. We divided these genomes into ones from a tree region with a modest phylogenetic dispersion (MD, 20 genomes) and those with a high phylogenetic dispersion (HD, 619 genomes) (Fig. 3b). Although the majority of genomes were not annotated at the species level, *Collinsella aerofaciens* was enriched in the HD group and other known *Collinsella* species were enriched in the MD group (Fig. 3c). SNV density in HD group was significantly higher than that of MD group (Fig. 3d). To test whether strain heterogeneity affects SNV density, we compared strain heterogeneity between the groups. We found that strain heterogeneity of HD group is significantly lower than that of non-*Collinsella* Actinobacteriaota species (*P* < 0.001 by Mann*–*Whitney *U* test) but not significantly different from that of MD group (Additional file 2: Fig. S8a). Given that SNV density of HD group is higher than that of non-*Collinsella* Actinobacteriaota species, this result suggests that strain heterogeneity is not associated with SNV density. Furthermore, we observed no correlation between strain heterogeneity and SNV density among all species that can be analyzed (Additional file 2: Fig. S8b), which also suggests that SNV density is not affected by strain heterogeneity.

SNV, a within-species genetic variation, is a major mechanism for the adaptation of commensal species to a distinct host environment. Wide dispersion of species branches indicates rapid speciation. Accordingly, high SNV density for a species with an overdispersed tree may indicate that the degree of within-species genetic variation may be positively associated with the speciation rate of gut commensals. To test this hypothesis, we examined the correlation between SNV density of 1521 representative species with ≥ 10 subspecies genomes and their phylogenetic distance to the five nearest species. The branch length to the neighboring species in the phylogenetic tree of a species that arose during rapid speciation tends to be short. We observed an inverse correlation between the average phylogenetic distance to the five nearest species and their SNV density (Fig. 3e), and a significantly higher SNV density for the top 10% species with shorter phylogenetic distance to the nearest five species than those for the bottom 90% of the 1521 species (Fig. 3f). This supports the model of a positive correlation of SNV density and the speciation rate of gut commensals.

### Functional landscape of 103 million proteins from human gut prokaryotes

Information on proteins encoded in the human gut microbes will facilitate the functional characterization of disease-associated microbiota. Using an in-house computational pipeline for cataloging human gut prokaryotic proteins (Additional file 2: Fig. S1c and Additional file 2: Fig. S9), we first identified 64,661,728 CDS (coding sequences) from 29,082 genomes from KIJ samples using Prodigal [36]. To reduce redundancy in the protein catalog, we first executed CD-HIT [37] at 100% similarity level and then combined with proteins cataloged by the UHGP-100 [11]. The consolidated protein catalog was next consecutively clustered by CD-HIT at lower sequence similarity levels: 95%, 90%, 70%, and 50%. This led to approximately 103.7, 20.0, 14.8, 8.5, and 4.7 million proteins at the sequence similarity levels of 100%, 95%, 90%, 70%, and 50%, respectively.

Unexpectedly, we observed that the UHGP contains proteins that are 100% identical, even in a catalog at 50% sequence similarity level. For instance, among the UHGP-50 proteins, GUT_GENOME232012_01109 and GUT_GENOME231777_00918 have an identical amino acid sequence. We identified 8663, 82,507, 243,362, and 75,620,150 proteins that are redundant at 100% similarity in the UHGP-50, UHGP-90, UHGP-95, and UHGP-100, respectively. Exclusion of the UHGP proteins that were 100% identical revealed that the HRGM contains more proteins than UHGP at all levels of sequence similarity except for 50% (Additional file 1: Table S5).

To facilitate the functional interpretation of gut microbiome profiles, we next annotated functional genomic elements and proteins in the HRMG. We predicted and annotated non-coding RNAs and functional peptides, using Prokka [30]; antibiotic resistance genes, using RGI [31] (Additional file 2: Fig. S10a); biosynthetic gene clusters, using antiSMASH [32] (Additional file 2: Fig. S10b); and 16S rRNA regions, using barrnap [26]. For functional annotation of proteins, we used eggNOG-mapper [38]. Notably, the landscape of antibiotic resistance ontology revealed that phylogenetically close species in the human gut tend to share antibiotic resistance mechanisms. A significantly large portion of the human gut prokaryotic proteins has not yet been functionally annotated. For the HRGM protein catalogs at 100%, 95%, 90%, 70%, and 50% similarity levels, 13.13%, 28.05%, 29.17%, 36.35%, and 47.62% of proteins, respectively, had no functional annotation, according to eggNOG-mapper. This effect appears to be amplified by redundant proteins, resulting in a reduced annotation rate at a low similarity level. Further, the annotation rate of proteins that are shared by many species is higher than that of species-specific proteins (Additional file 2: Fig. S11).

### HRGM improves taxonomic and functional classification of sequencing reads

**Fig. 4:**
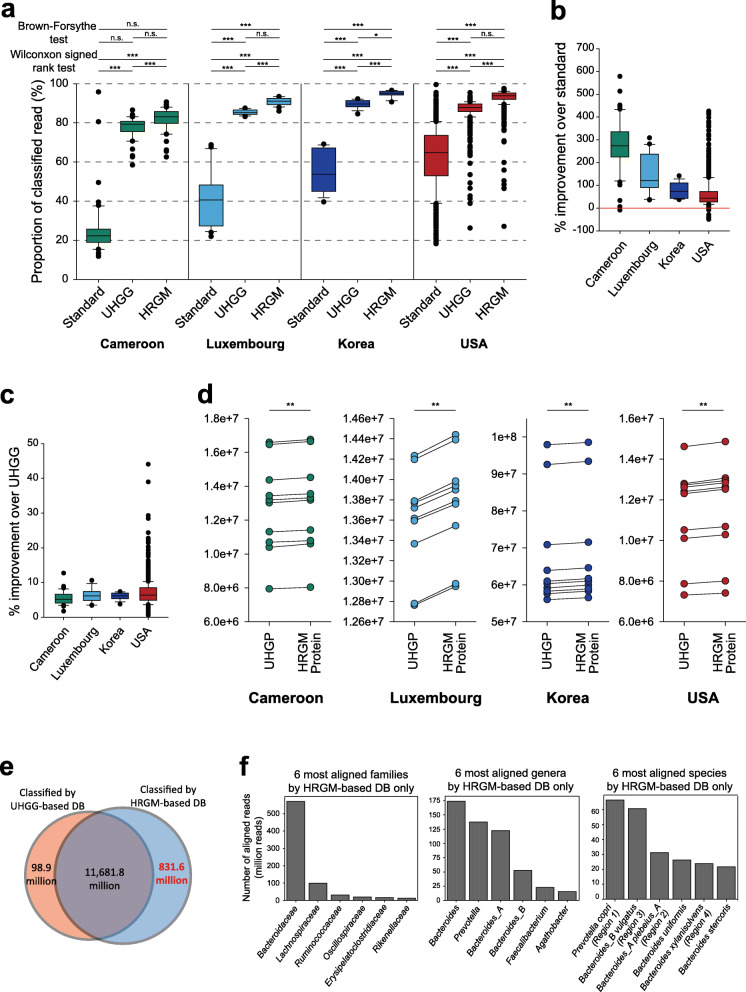
Improvement of taxonomic and functional classification of sequencing reads by HRGM. **a** Proportion of taxonomically classified sequencing reads of WMS data from four different populations. The significance of the improvement was calculated by Wilcoxon signed-rank test. Brown–Forsythe test was used to evaluate the decrease of variance. **b, c** Percent improvement of the read classification proportion in HRGM-based database compared with the standard database (**b**) and the UHGG-based database (**c**). **d** The number of reads aligned to the UHGP-95 and HRGM-95 protein catalogs. Statistical significance was calculated by using Wilcoxon signed-rank test. **e** The number of sequencing reads that are classified by UHGG and HRGM-based kraken2 database. **f** Top six most aligned families (left), genera (center), and species (right) by 831.6 million reads that are classified by HRGM-based database but not by UHGG-based database

According to a recent benchmark study, whole-DNA–based methods outperform marker-based methods for taxonomic classification of metagenomic sequencing reads [58]. The performance of whole-DNA–based methods relies on the quality of the reference genome database. The standard databases lack numerous genomes of species that exist in the human gut, which leads to false-negatives, while including many genomes from other microbial communities, which leads to false-positives [58]. We hypothesized that the HRGM, which is specific to the human gut microbiome and more comprehensive than other databases, can improve the taxonomic classification of sequencing reads. We used Kraken2 [43] to compare the taxonomic classification of three genome databases: a standard database that contains RefSeq [59] complete genomes (RefSeq CG) of bacterial, archaeal, and viral domains; the UHGG-based database containing 4644 representative genomes; and the HRGM-based database containing 5414 representative genomes. To generate independent test datasets, we compiled WMS data for 1022 fecal samples from the USA, Cameroon, Luxembourg, and Korea, which were not included in the UHGG nor HRGM. We then evaluated the efficacy of Kraken2 classification based on the proportion of classified reads (“Methods”). The average classification efficacy using the UHGG and HRGM-based databases was 44.6% and 54.4% higher, respectively, than that of the standard database (Fig. 4a-b, *P* < 0.001, two-sided Wilcoxon signed-rank test). In addition, the variance of the read classification rate of custom databases was significantly smaller than that of the standard database, except for the Cameroon population (Fig. 4a, *P* < 0.001, Brown*–*Forsythe test). Importantly, the read classification efficacy of the HRGM-based database was significantly improved by 6.9% on average compared with that of the UHGG-based database for the four test samples (Fig. 4a and c, *P* < 0.001, two-sided Wilcoxon signed-rank test), which suggests that the updated reference genome database improves taxonomic classification of the gut metagenomic sequencing data.

Next, we investigated the efficacy of functional classification based on the number of aligned sequencing reads from reference protein databases. Because of the extremely large number of reference proteins, we used only 40 samples randomly selected from the 1022 fecal samples (10 samples from each population), and aligned the sequencing reads with the UHGP-95 and HRGM-95 protein catalogs (Methods). The number of aligned reads was 1.31% higher, on average, with HRGM-95 in all tested samples than with UHGP-95 (Fig. 4d), although HRGM-95 contains 0.4% more proteins than UHGP-95.

Searching for the factors that contribute to the improved classification of sequencing reads, we compared classified reads by UHGG- and HRGM-based databases. Among a total of 12,612.3 million reads aligned by any of the databases, 11,681.8 million reads (92.6%) were classified by both databases. The number of reads aligned by HRGM-based database only (831.6 millions) was 8.4-fold larger than that by UHGG-based database only (98.9 millions) (Fig. 4e). We then investigated the highest contributing taxa to the 831.6 million reads aligned by HRGM-based database only (i.e., the most aligned taxa by the 831.6 million reads). We found *Bacteroidaceae* as a dominant contributing family and *Bacteroides, Prevotella*, *Bacteroides_B*, and *Bacteroides_A* as the top four contributing genera (Fig. 4f, panels on the left and center). These results are consistent with our observation that *Bacteroidaceae* family was enriched for the new species from KIJ samples (Fig. 2a) and that the majority of its species belong to the *Bacteroides* and *Prevotella* genera (Fig. 2b). Notably, the top contributing species taxa also were the regions of the phylogenetic tree that were enriched for the new species: Region 1 (*Prevotella copri*), Region 2 (*Bacteroides_A plabeius_A*), Region 3 (*Bacteroides_B vulgatus*), and Region 4 (*Bacteroides xylanisolvens*) (Fig. 4f, right panel). Therefore, these results suggest that the MAGs for the new 780 species from under-represented Asian countries were the main contributors to the improved taxonomic and functional classification of sequencing reads.

### Deep sequencing is recommended for profiling low-abundance taxa

**Fig. 5:**
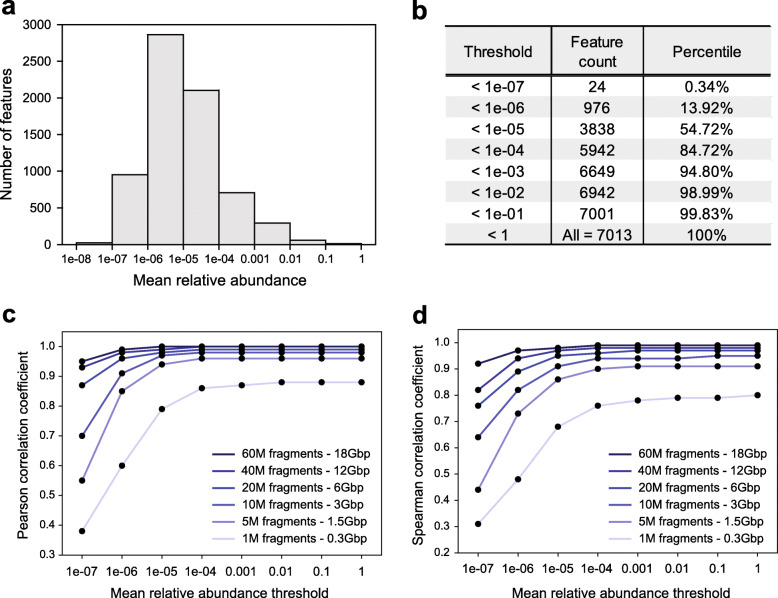
*Effect of sequencing depth on the reliability of taxonomic profiles.***a** The distribution of taxonomic features over different mean relative abundances. **b** The cumulative proportion of taxonomic features at different thresholds of mean relative abundance. **c, d** Pearson correlation coefficient (*PCC*) (***c***) and Spearman correlation coefficient (*SCC*) (***d***) of the taxonomic profiles at the given sequencing depth and 80 M fragments. The *x*-axis (the mean relative abundance threshold) indicates the upper boundary of the mean relative abundance

In a previous study, taxonomic profiles obtained by shallow sequencing (0.5–2 million reads) showed a high correlation with those obtained by ultra-deep sequencing (2.5 billion reads) [60]. However, this evaluation was based on entire taxa, in which highly abundant or core taxa govern the correlation measure. Further, low-abundance taxa likely play important, as yet unknown, biological roles in the gut microbial communities [61, 62]. We therefore evaluated the impact of sequencing depth on the reliability of taxonomic profiling for different ranges of taxon abundance. We generated simulated datasets at various sequencing depths using WMS data for 16 new Korean fecal samples that were not included in the HRGM. We then stratified the taxonomic features into eight different groups, according to the mean relative abundance (Fig. 5a, b). We calculated the mean *PCC* and the mean *SCC* between the taxonomic profiles at different sequencing depths for different mean relative abundances (“Methods”). The taxonomic profile similarity between two groups showed increasing *PCC* and *SCC* with an increasing sequencing depth. For example, > 10 million read pairs (3 Gbp) may be needed for taxonomic profiles that highly correlate (*PCC* > 0.9) with those based on 80 million read pairs (25 Gbp) to account for the features with the lowest 13.92% of relative abundance (relative abundance <1e−06) (Fig. 5c and Additional file 2: Fig. S12a). For *SCC* > 0.9, the required sequencing depth increased to 20 million read pairs (6 Gbp) for taxonomic features with a similar level of relative abundance (Fig. 5d and Additional file 2: Fig. S12b). Overall, these observations based on Korean gut metagenomes suggest that deep sequencing (e.g., > 20 million read pairs) may be needed for reliable taxonomic profiles of low-abundance taxa.

### Bacterial taxa with high cross-reactivity potential may promote inflammation

**Fig. 6:**
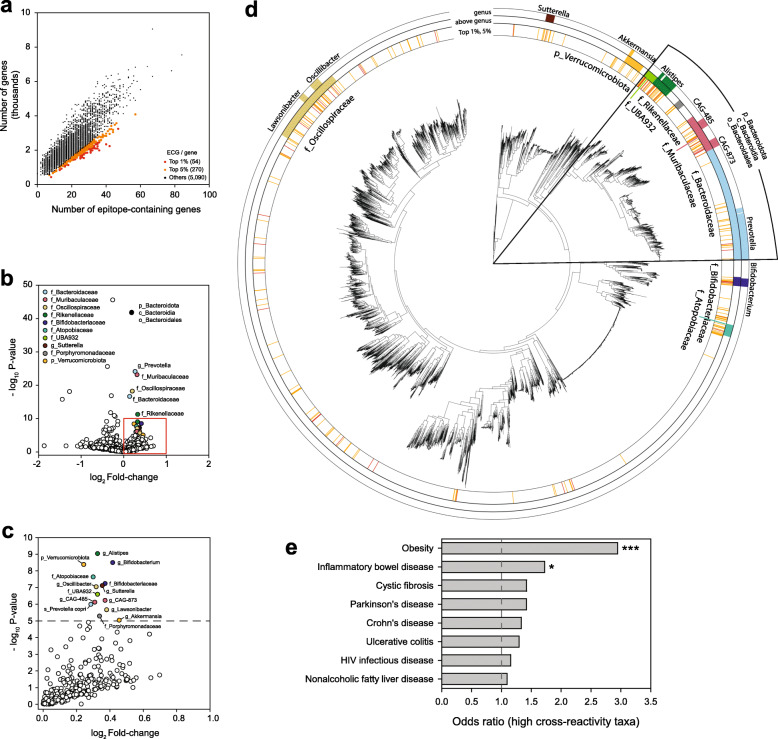
Analysis of cross-reactivity potential of gut bacterial taxa. **a** The number of genes and autoimmune epitope-containing genes (ECGs) in 5414 genomes of species representatives. Red and orange points, species with the top 1% and 5% ECG per gene, respectively. **b** Volcano plot of the enrichment of ECG density. Taxonomic clades with positive log2 fold-change and *P* < 1e−5 are highlighted with different colors. Taxonomic clades denoted by the same color have an inclusive relationship (e.g., *g_Prevotella* belongs to *f_Bacteroidaceae*), with the exception of p_Bacteroidota, c_Bacteroidia, and o_Bacteroidales. The first character of each clade name indicates the taxonomic levels (p: phylum; c: class; o: order; f: family; and g: genus). **c** The red-highlighted area from **b**. **d** Maximum-likelihood phylogenetic tree with taxonomic annotations of clades with high ECG density. The first layer represents clades with the top 1% (red) and 5% (orange) ECG density [annotations and color designations are the same as in **a**]. The second and third layers represent enriched taxonomic clades in the volcano plot [taxonomic annotations and color designations are the same as in **b** and **c**]. The second layer represents above-genus-level annotations. The third layer represents genus-level taxonomic clades. **e** Odds ratio (odds that high cross-reactivity taxa are associated with the disease / odds that low cross-reactivity taxa are associated with the disease) for diseases with ≥ 40 annotated taxa by gutMDisorder database. Statistical significance was calculated by Fisher’s exact test (*: *P* < 0.05; ***: *P* < 0.005)

Microbial peptides homologous to the host self-antigens may stimulate immune cells and, hence, the hypothesis of molecular mimicry has emerged as a mechanism underlying autoimmune diseases [63]. To systematically evaluate the contribution of cross-reactive microbial antigens to human diseases, we analyzed human self-antigen homologs on the HRGM species genomes. We first compiled epitope sequences involved in autoimmune diseases from the IEDB [49], and then we used them for homology-searches against 5414 representative species genomes to find ECGs (“Methods”). We found that these ECGs were enriched for core functions that were highly conserved between bacteria and human. For example, “enolase (K01689)” which exists in both bacterial species and humans was enriched among ECGs by > 544-fold compared with non-ECGs (Additional file 1: Table S13). This result can be explained by the fact that gut commensal bacterial proteins with high sequence homology to host human proteins may have high probability of harboring cross-reactive peptides. We also accessed enrichment of TMHs and signal peptides for ECGs and found that TMHs were depleted in ECGs (odds ratio = 0.784 and *P* value = 5.43e−228 by Fisher’s exact test), whereas signal peptides were enriched in ECGs (odds ratio = 1.171 and *P* value = 1.23e−71 by Fisher’s exact test). Next, we identified bacterial species with high cross-reactivity potential based on the density of the encoded cross-reactive epitopes. Because the number of ECGs increases as the number of coding genes increases (Fig. 6a), we divided the ECG count by the total number of genes for each species. Some human gut commensals had a relatively high cross-reactivity potential (Fig. 6b, c, “Methods”). On the genus level, *Akkermansia*, *Alistipes*, *Bifidobacterium*, *Lawsonibacter*, *Oscillibacter*, *Prevotella*, and *Sutterella* have a high cross-reactivity potential (Fig. 6d). All other taxa were defined as low cross-reactivity taxa. We systematically evaluated association between the high cross-reactivity taxa and each disease, based on taxon-disease pairs annotated by gutMDisorder database [51]. We then examined the direction of the association (tendency to increase or decrease of the high cross-reactivity taxa in the disease). Among human diseases with ≥ 40 associated taxa by gutMDisorder, we found that obesity and inflammatory bowel disease were significantly more associated with high cross-reactivity taxa than to low cross-reactivity taxa (Fig. 6e, Additional file 1: Table S14). Presumably, cross-reactive bacterial antigens induce inflammation, which is an underlying pathogenic mechanism in not only inflammatory bowel disease but also in obesity [64]. This suggests that bacterial taxa with high cross-reactivity potential may contribute more to the pathogenesis of gut microbiome-associated diseases than those with low cross-reactivity potential by promoting inflammatory conditions. We also found that high cross-reactivity taxa tend to decrease in both associated diseases, which may be explained by the action of immune cells with specificity to the cross-reactive antigens.

## Discussion

In the present study, we constructed an improved catalog of the human reference gut prokaryotic genomes and their proteins, by including MAGs from fecal metagenomes from under-represented Asian countries. Inclusion of the newly assembled genomes expanded the catalog size by over 10%. In addition, we demonstrated that database expansion also significantly improved the taxonomic and functional classification of sequencing reads. Many new species obtained from this study were associated with diet-related lifestyles at the sampled geographic locations. Therefore, complementation of metagenome datasets to account for under-sampled geographical locations and lifestyles might be an effective strategy for improving the human reference gut microbiome.

We also demonstrated that the analysis of microbial DNA and peptide sequences facilitates the understanding of gut commensal speciation and interactions with the host immunity. The colonizing commensal microbes adjust to their host environment via genetic changes and selection, which lead to genetic variation within species. We cataloged the SNVs of conspecific genomes and found that the SNV density of gut prokaryotic species is inversely correlated with the phylogenetic distance to their neighboring species. This may suggest that the degree of within-species genetic variation is positively associated with the speciation rate of gut commensal microbes. Whether SNV actually enhances the speciation rate should be addressed in future investigations. Finally, we showed that gut bacterial taxa with high cross-reactivity potential are more associated with pathogenic inflammatory conditions than are those with low cross-reactivity potential through systematic analysis of microbial peptide sequences homologous to the host auto-antigens. Such analysis is only possible if microbial protein sequences are available with the corresponding taxonomic information.

As the WMS analysis for population-wide human gut microbiome profiling increases in popularity, the choice of sequencing depth is an important factor to consider in study design. In the present study, we found that deep sequencing (e.g., > 20 million read pairs) was necessary for reliable taxonomic profiling of low-abundance commensals in the Korean gut microbiome samples. The current knowledge of human gut microbiome is biased towards core taxa that are usually highly abundant. Low sequencing depth (e.g., 0.5–2 million read pairs) may be sufficient for the profiling of core taxa, but not those with low abundance. Deep sequencing may therefore be needed for the WMS-based analysis of human gut microbiome to investigate the function of relatively unexplored low-abundance species. However, there are additional factors that affect recovery of taxa other than sequencing depth. For example, the complexity of a metagenome that also affects the efficacy of taxonomic profiling substantially varies between individuals, disease status, and ages. Accordingly, the results from this study may provide guidelines for the choice of sequencing depth for the analysis of human gut microbiome in future studies.

## Conclusions

In summary, by including gut metagenomes of previously under-represented Asian countries, Korea, India, and Japan, we developed a substantially expanded microbiome catalog, HRGM. Sequence data and functional information for 232,098 non-redundant genomes of 5414 representative prokaryotic species along with protein sequences and SNVs will be available from a web server (www.mbiomenet.org/HRGM/). We will periodically update the genome and protein catalogs as new WMS data for new fecal samples become available. HRGM will provide a versatile resource for functional dissection of disease-associated gut microbiota.

## Supplementary Information

**Additional file 1.** Supplementary Tables.

**Additional file 2.** Supplementary Figures.

## Data Availability

Raw metagenomic sequencing data are available from the NCBI Sequence Read Archive (SRP292575) https://trace.ncbi.nlm.nih.gov/Traces/sra/?study=SRP292575 [65]. The MAGs assembled from KIJ samples were also deposited to NCBI Genome (PRJNA678426, PRJNA730993) https://www.ncbi.nlm.nih.gov/bioproject/PRJNA678426/; https://www.ncbi.nlm.nih.gov/bioproject/PRJNA730993/ [66, 67]. By accessing the web server, www.mbiomenet.org/HRGM/ [68], users can browse and download all genomes for 5414 representative species, their annotations, and metadata, including geographical origin, taxonomy, genomic content, and genome statistics. The five classes of protein catalogs, 16S rRNA sequences, and SNVs are also provided with their functional annotation and taxonomic origin. Other publicly available data used in this project: Whole-metagenomic shotgun sequencing data from Dhakan et al. 2019 is deposited under Bioproject: PRJNA397112 [12]. Whole-metagenomic shotgun sequencing data from Yachida et al. 2019 is deposited under Bioproject: PRRJDB4176 [13]. UHGG and UHGP catalogs are available at https://www.ebi.ac.uk/metagenomics/genomes [11].
