# Supplementary material for: Human reference gut microbiome catalog including newly assembled genomes from under-represented Asian metagenomes
Source: Genome Med. 2021 Aug 27;13:134. doi: 10.1186/s13073-021-00950-7 (PMC8394144; doi:10.1186/s13073-021-00950-7)
Supplement: Supplementary file 2 — Additional file 2. Supplementary Figures. [file 13073_2021_950_MOESM2_ESM.docx]

***Supplementary Figures***

**Human reference gut microbiome catalog including newly assembled genomes from under-represented Asian metagenomes**

Chan Yeong Kim, Muyoung Lee, Sunmo Yang, Kyungnam Kim, Dongeun Yong, Hye Ryun Kim, and Insuk Lee

**Fig. S1-12**


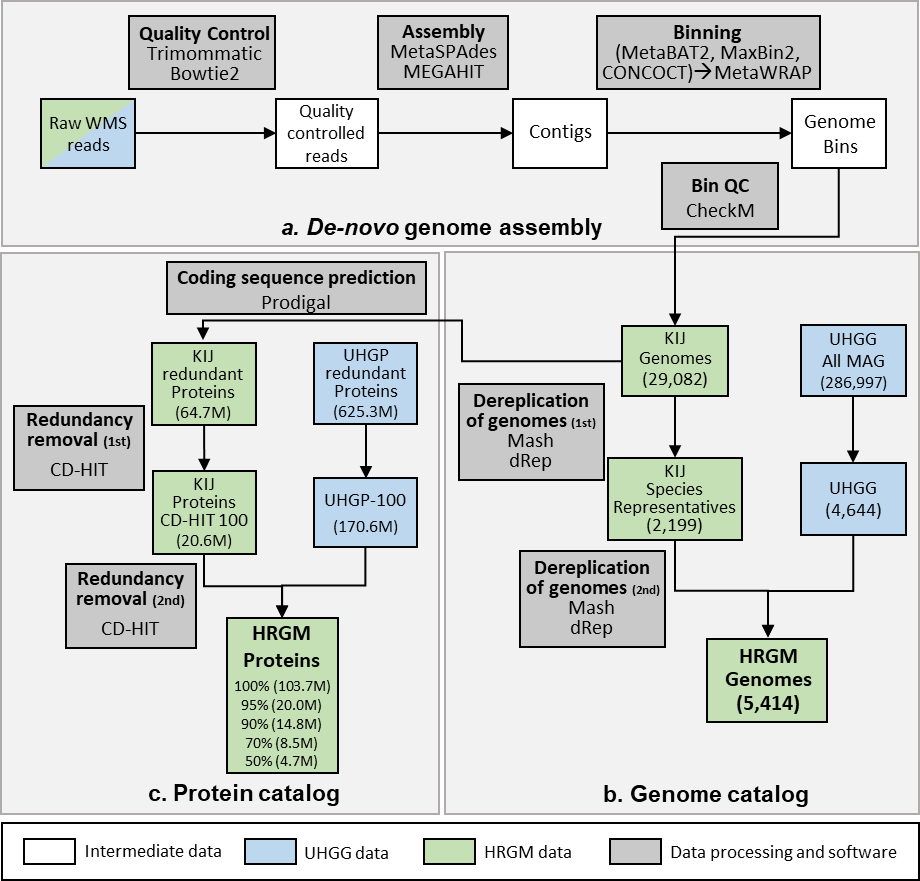


**Fig. S1 | Overview of computational pipeline for cataloging genomes and proteins from whole metagenomic shotgun sequencing data.**


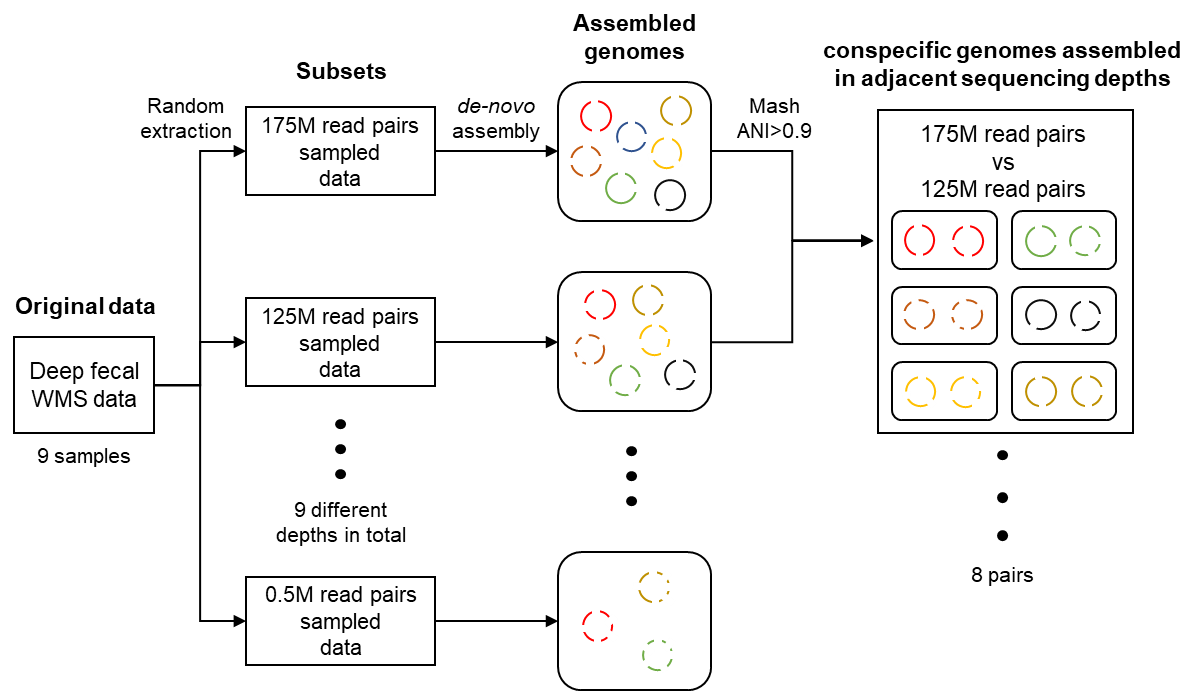


**Fig. S2 | Overview of generating simulated data sets for assessing effect of sequencing depth on *de novo* genome assembly.** Flow diagram that describes methods of assessing the effect of sequencing depth on genome assembly with simulated data. We performed a *de novo* genome assembly on 81 simulated samples (9 depth-levels from 9 original samples). Genomes with the same color indicate that the genomes originated from the same species.


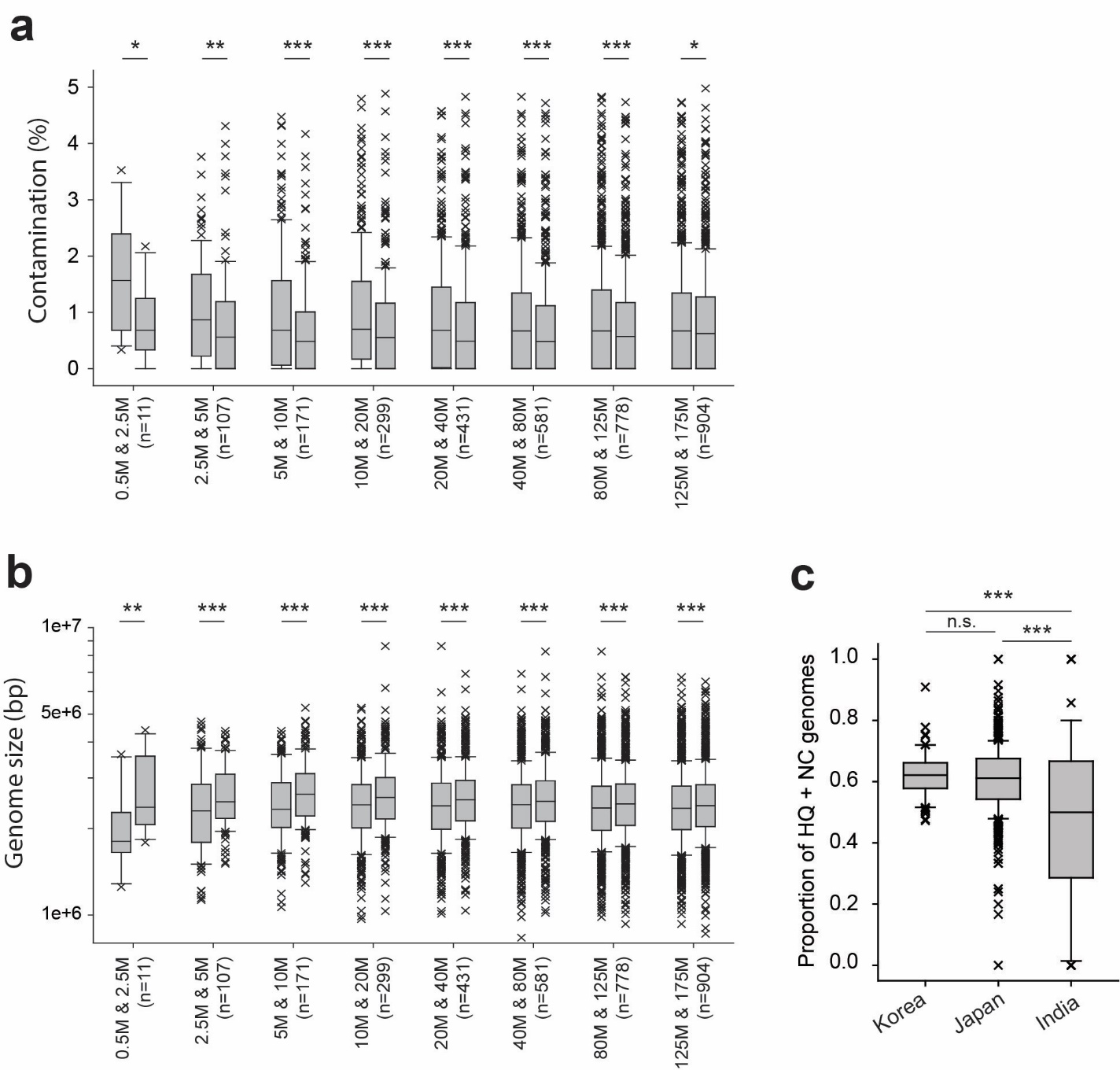


**Fig. S3 | Quality of assembled genomes increases as sequencing depth increases.** The quality of the same genome assembled from different sequencing depth was evaluated by **a.** contamination, **b.** size of the genome. **c.** The proportion of high-quality (HQ + NC) genomes assembled from Korea, Japan, and India. *P*-values were evaluated by the two-sided Mann-Whitney U test. (n.s: *P* > 0.05, *: *P* < 0.05, **: *P* < 0.01, ***: *P* < 0.001)


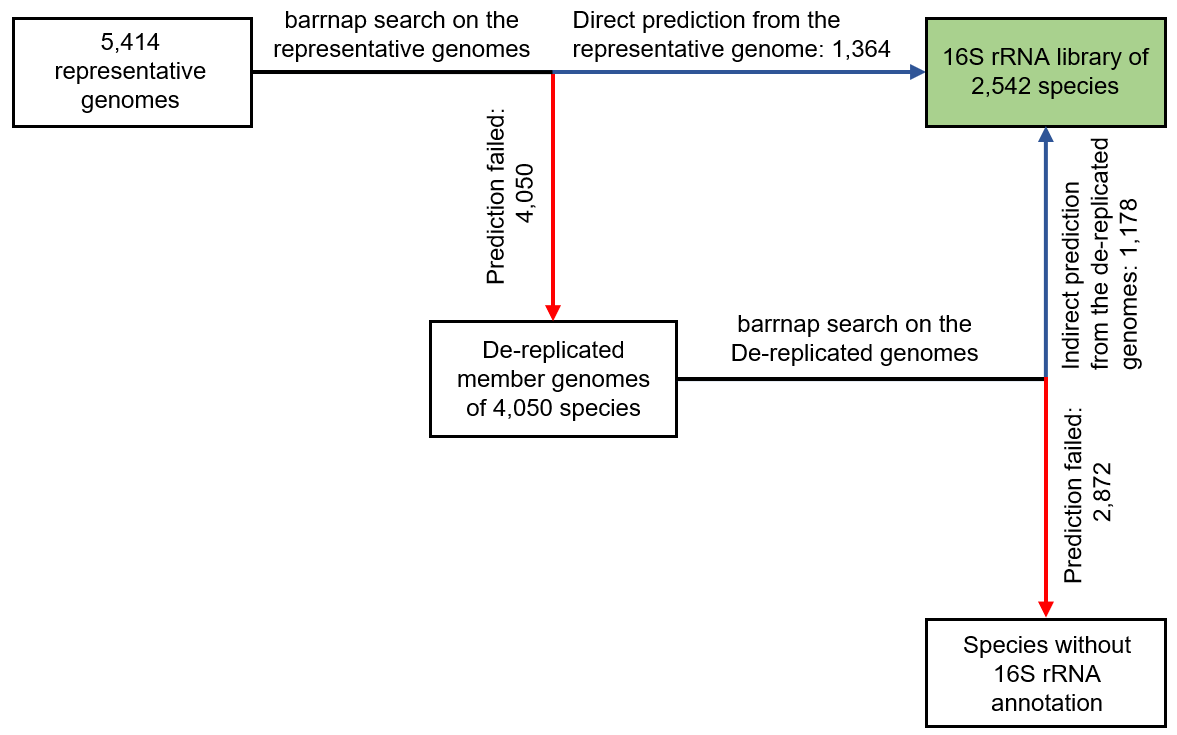


**Fig. S4 | Computational pipeline for predicting 16S rRNA sequence region.** 16S rRNA regions were predicted with barrnap from representative genomes, and from de-replicated member genomes when failed to predict from representative genomes. Blue arrows represent the success of barrnap prediction, and red arrows represent the failed prediction.

**
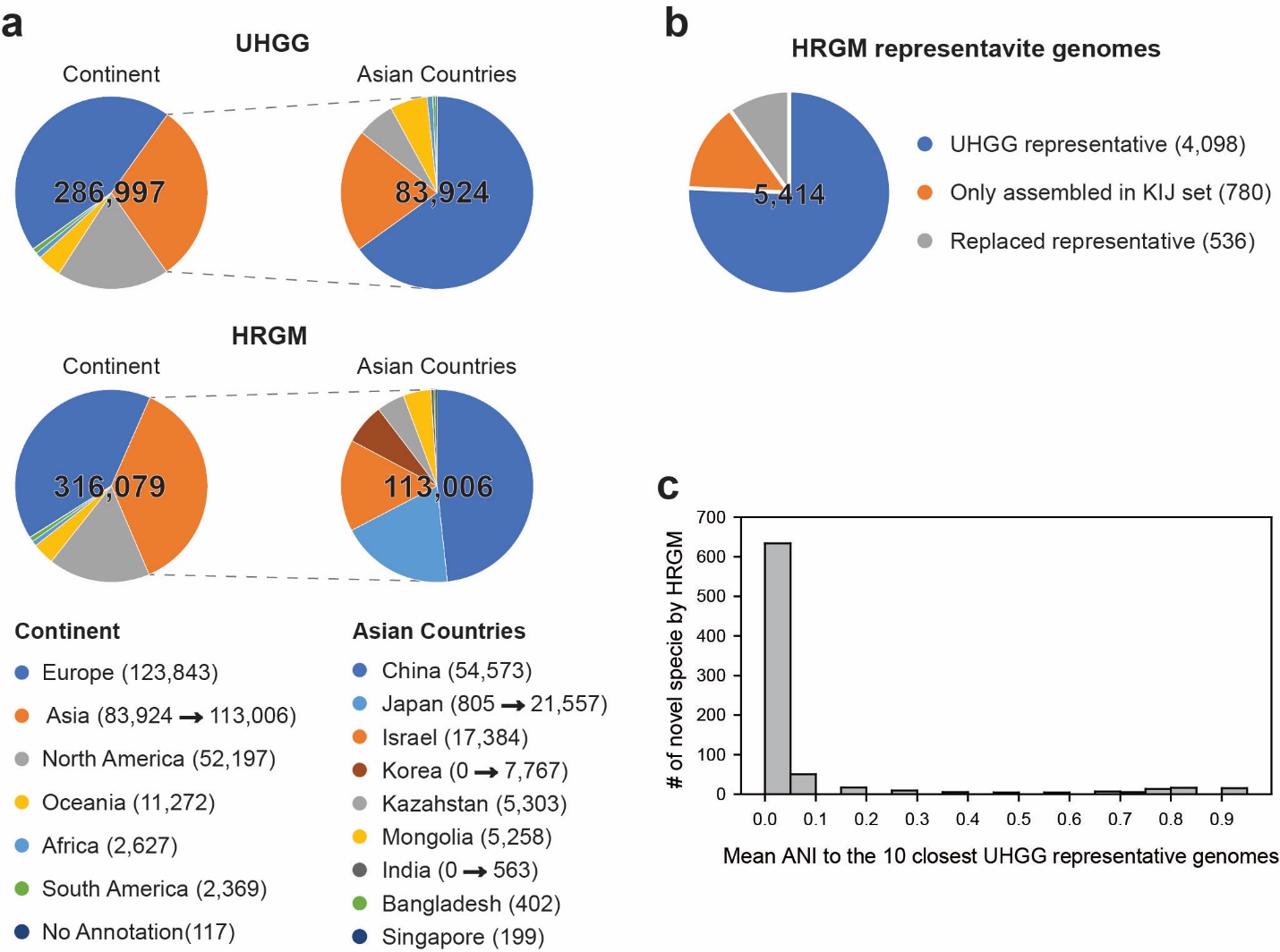
**

**Fig. S5 | Comparison between UHGG and HRGM a,** Pie charts representing the number of MAGs by continents (left) and Asian countries (right) of UHGG (upper) and HRGM (lower) genome catalog. The exact number of MAGs for each continent and country is represented in the below legend. Arrows represent the change of MAGs from UHGG to HRGM. **b,** The number of representative genomes by their originated datasets. **c,** Distribution of mean average nucleotide identity (ANI) of 780 new species genomes by HRGM to the 10 closest UHGG species representative genomes.

**
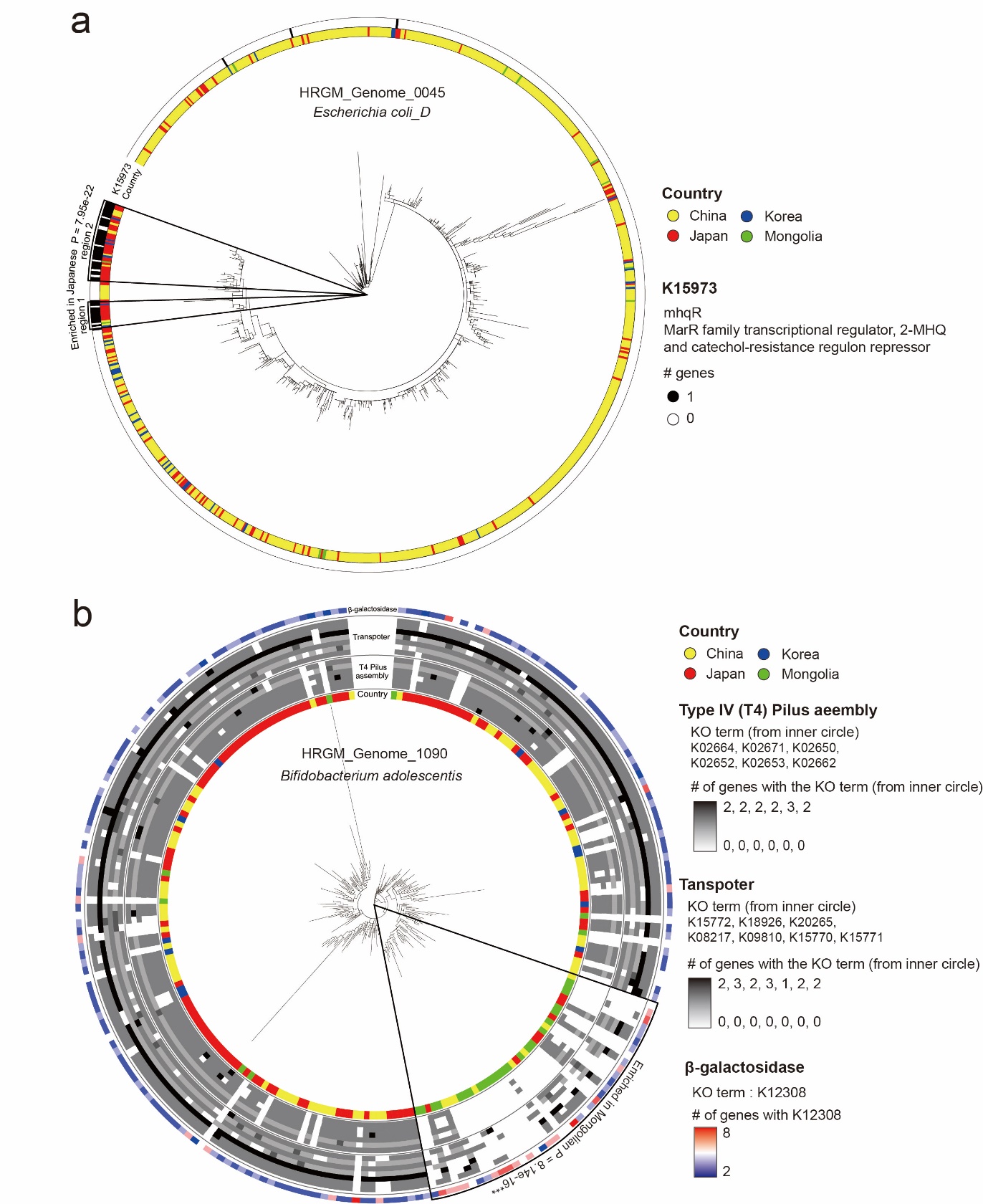
**

**Fig. S6 | Phylogenetic tree of species that contain endemic subspecies to a specific Asia country** (a) A clade of Escherichia coli D subspecies that are enriched for MarR orthologs are endemic to Japan. (b) A clade of Bifidobacterium adolescentis subspecies that are depleted for orthologs of Type IV pilus assembly genes and transporters are endemic to Mongolia.

a


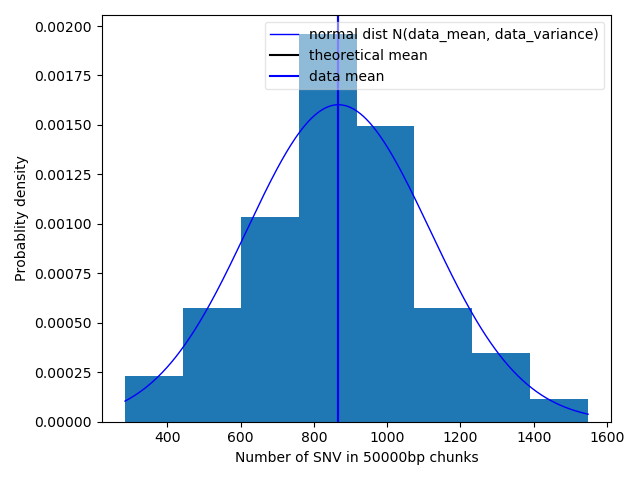


b


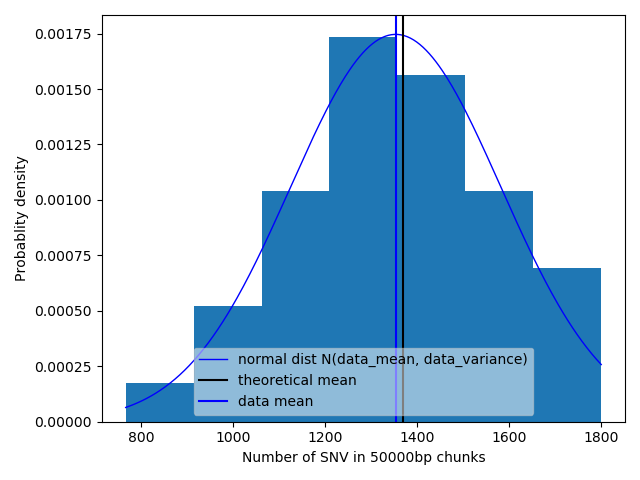


**Fig. S7 | Distribution of the number of SNVs in chunks (fragments) of genomes***.* The histogram represents the distribution of # SNVS in *r-g* pairs. **a.** *r* = HRGM_Genome_1867, *g* = KIJ_genome_5221, **b.** *r* = HRGM_Genome_2859, *g* = KIJ_genome_719. Blue and black vertical bars represent theoretical and observed mean of data. Blue curve is a normal distribution with empirical mean and standard deviation.


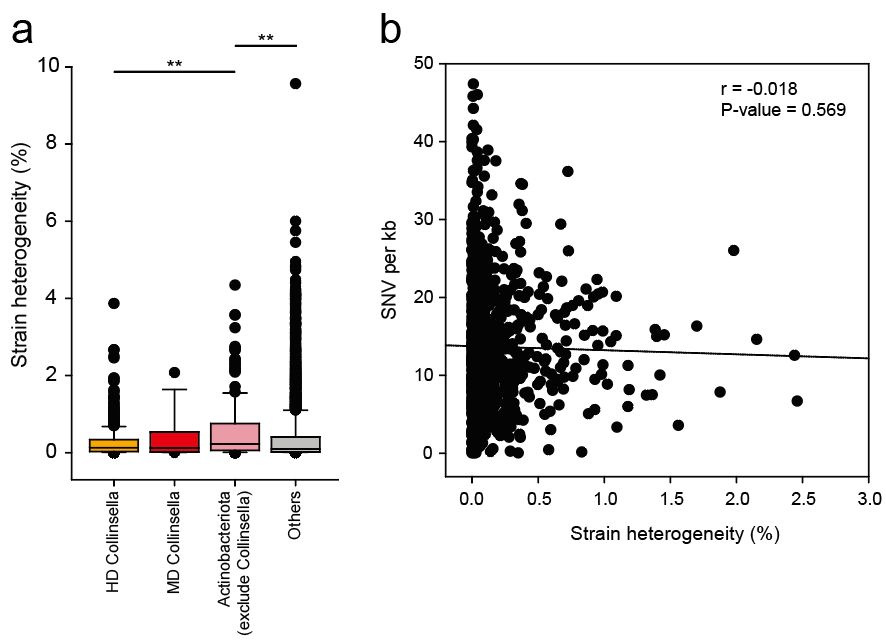


**Fig. S8 | Evaluation of relationship between Strain heterogeneity and SNV density**. **a.** Strain heterogeneity (analyzed by CMseq) of HD Collinsella, MD Collinsella, non-Collinsella Actinobacteriota and other species (**: P-value < 0.001 by Mann Whitney U test) **b**. Correlation between strain heterogeneity and SNV density.

**
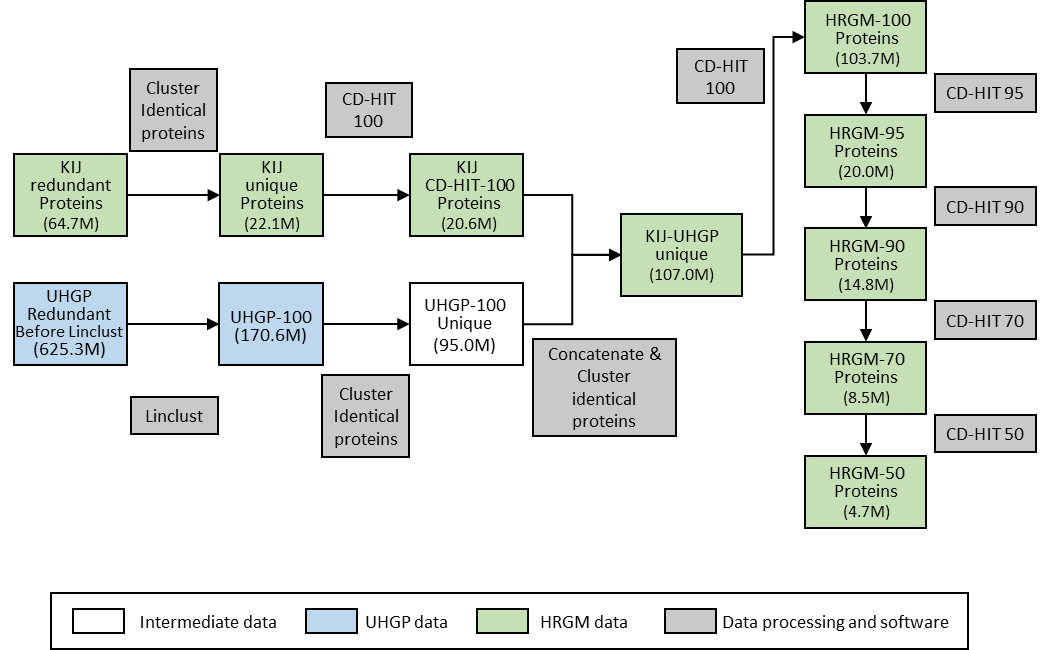
**

**Fig. S9 | Overview of computational pipeline for cataloging nonredundant proteins.** The number in the parentheses indicates the number of proteins after each step. Information of proteins for the green boxes are freely available.

**
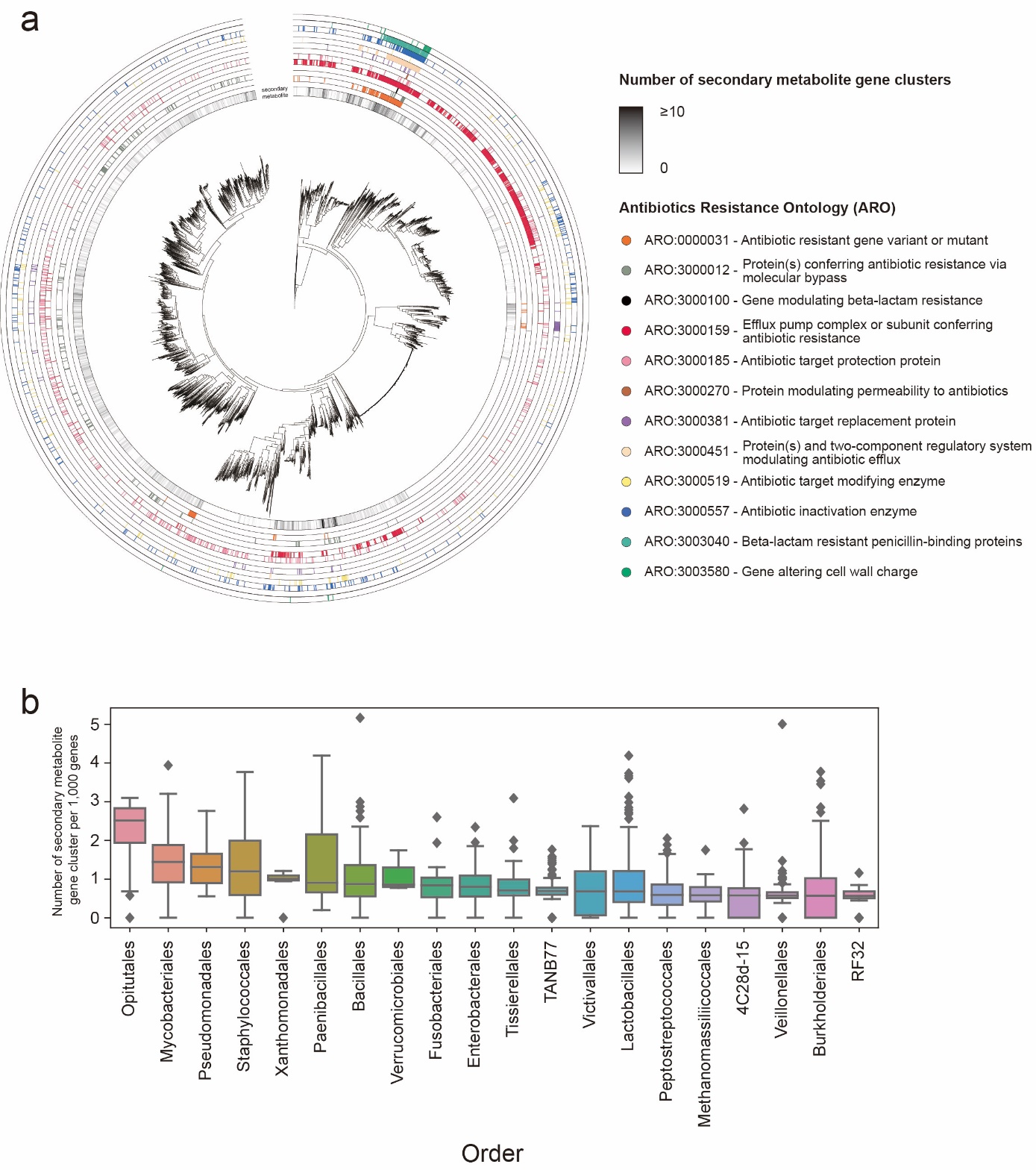
**

**Fig. S10 | The landscape of secondary metabolite gene clusters and antibiotics resistance ontology of human gut prokaryotic species.** (a) The number of secondary metabolite gene clusters, identified by antiSMASH, for each genome is indicated by grayscale gradient. Genomes having antibiotic resistance gene(s) are annotated according to ARO (antibiotic Resistance Ontology) terms. 12 labels are third level ARO terms which are annotated by at least one genome for each of the 5,414 HRGM representatives. (B) The number of secondary metabolite gene cluster normalized by the gene count of each order.

**
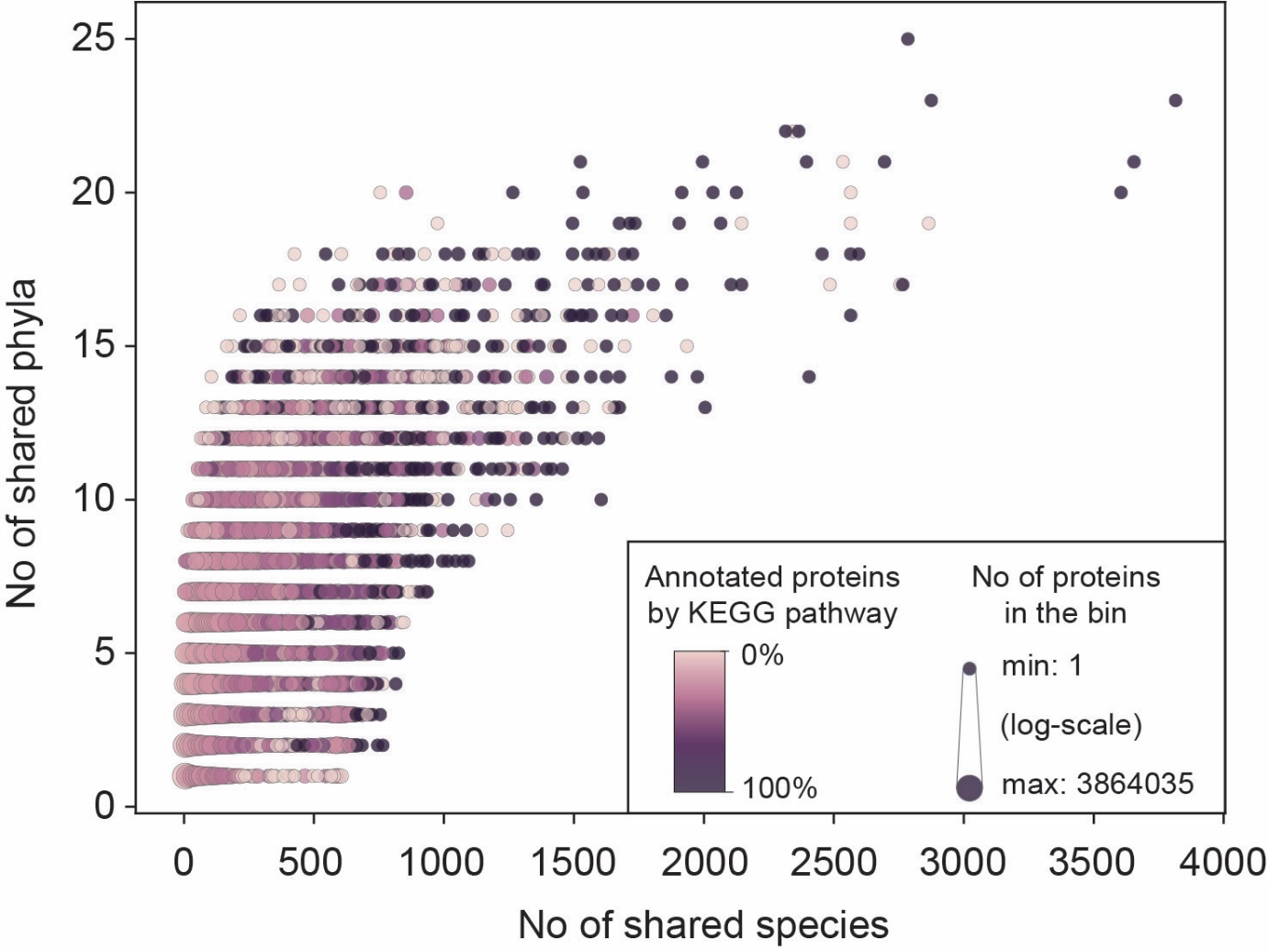
**

**Fig. S11 | Gut microbial proteins that are shared by many species tend to be functionally annotated.**Binned scatter plot that represents the annotation rate of each protein bin. Proteins of the HRGM Protein-50 catalog were sorted by the number of their encoding species (X-axis) and annotation rates were measured for every bin of 10 proteins. Bright color indicates the proteins poorly annotated by the KEGG pathway. The size of data point represents the number of proteins in each bin.

**
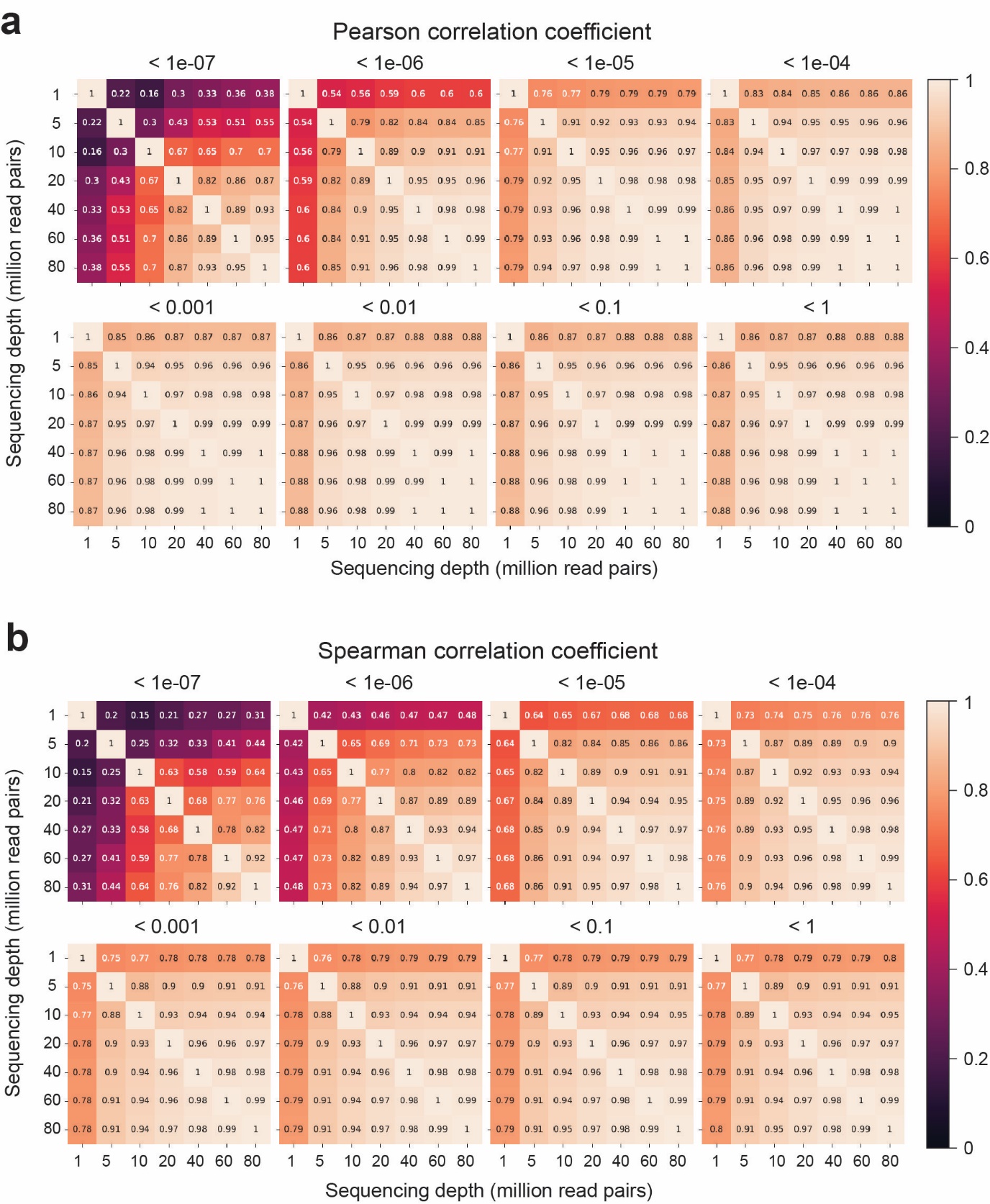
**

**Fig. S12 | The effect of sequencing depth on taxonomic profiles based on WMS data**. **a**, Pearson correlation coefficient and **b**, Spearman correlation coefficient of the taxonomic profiles between the various sequencing depths (x- and y- axis) for the given mean relative abundance thresholds (title of each heatmap).
